# Genome-wide mapping of somatic mutation rates uncovers drivers of cancer

**DOI:** 10.1038/s41587-022-01353-8

**Published:** 2022-06-20

**Authors:** Maxwell A. Sherman, Adam U. Yaari, Oliver Priebe, Felix Dietlein, Po-Ru Loh, Bonnie Berger

**Affiliations:** 1 Computer Science and Artificial Intelligence Laboratory, Massachusetts Institute of Technology, Cambridge, MA USA; 2 Harvard-MIT Health Sciences and Technology Program, Cambridge, MA USA; 3 Division of Genetics, Department of Medicine, Brigham and Women’s Hospital and Harvard Medical School, Boston, MA USA; 4 Broad Institute of MIT and Harvard, Cambridge, MA USA; 5 The Center for Brains, Minds and Machines of MIT and Harvard, Cambridge, MA USA; 6 Department of Physics, University of Pennsylvania, Philadelphia, PA USA; 7 Department of Medical Oncology, Dana-Farber Cancer Institute, Harvard Medical School, Boston, MA USA; 8 Department of Mathematics, Massachusetts Institute of Technology, Cambridge, MA USA; 9 Present Address: Computational Health Informatics Program, Boston Children’s Hospital, Boston, MA USA

**Keywords:** Cancer genomics, Machine learning

## Abstract

Identification of cancer driver mutations that confer a proliferative advantage is central to understanding cancer; however, searches have often been limited to protein-coding sequences and specific non-coding elements (for example, promoters) because of the challenge of modeling the highly variable somatic mutation rates observed across tumor genomes. Here we present Dig, a method to search for driver elements and mutations anywhere in the genome. We use deep neural networks to map cancer-specific mutation rates genome-wide at kilobase-scale resolution. These estimates are then refined to search for evidence of driver mutations under positive selection throughout the genome by comparing observed to expected mutation counts. We mapped mutation rates for 37 cancer types and applied these maps to identify putative drivers within intronic cryptic splice regions, 5′ untranslated regions and infrequently mutated genes. Our high-resolution mutation rate maps, available for web-based exploration, are a resource to enable driver discovery genome-wide.

## Abstract (web summary)

Cancer driver mutations are identified by predicting neutral mutation rates across the entire genome.

## Main

Neutral (passenger) mutations that do not provide a proliferative advantage to a cell dominate the mutational landscape of tumors^1,2^. Only a relatively small fraction of mutations are under positive selection^3–5^ due to their ability to drive cancer by promoting cell growth, resisting cell death or enabling tissue invasion^6^. Because positively selected mutations reoccur across tumors^7^, genomic elements (for example, coding sequences, promoters, enhancers and long non-coding RNAs) with carcinogenic potential accumulate more mutations than expected compared to the rates at which neutral mutations occur when counted across multiple tumors^8,9^. Searching for mutational excesses attributable to positive selection to discover driver mutations, genes and non-coding elements provides crucial insight into the mechanisms of cancer^4,5,10–15^.

Because robust identification of mutational excess requires an accurate model of the neutral mutation rate, computational tools that carefully model somatic mutation rates are central to locating additional cancer drivers. This task is made challenging by the highly variable and tissue-specific patterns of neutral mutations across the cancer genome^16,17^. Existing methods address this challenge by fitting bespoke statistical models of mutation rates to specific regions of the genome^4,9,18–21^. For example, methods designed to identify driver genes model mutation rates specifically within protein-coding sequences by using synonymous mutations as a proxy for neutral mutations^3,4,21,22^. Recent methods designed to identify non-coding cancer drivers train sophisticated machine learning methods, such as gradient boosting machines, to model mutation rates within a subset of the genome^18–20^ (~4% of the genome in a recent pan-cancer analysis of non-coding drivers^5^). Additionally, some models search for driver mutations in unexpected nucleotide contexts^10^, in unexpected clusters^23^ or by directly (and interpretably) predicting the consequences of variants within the coding sequence of select genes^24^. Despite this progress, the ability to search for evidence of driver mutations in arbitrary genomic regions remains incomplete: existing methods are not applicable to most of the genome (for example, because they operate only within coding sequences); require time-consuming and computationally expensive model training for each set of regions to test in a cancer cohort; or cannot test with base-pair resolution. These limitations contribute to catalogs of cancer driver elements remaining incomplete, particularly in the non-coding genome^25^, hindering precision oncology^4,11,26,27^.

Here we introduce a genome-wide neutral mutation rate model that allows rapid testing for evidence of positively selected driver mutations anywhere in the genome. This approach, called Dig, is predicated on two key methodological advances. First, we introduce a deep learning approach to map cancer-specific somatic mutation rates at kilobase-scale resolution across the entire genome. Second, we propose a probabilistic model that uses these maps to test any set of candidate mutations from an arbitrary cancer cohort for evidence of positive selection. Through this framework, our maps enable millions of mutations to be evaluated in arbitrary cancer cohorts in minutes using the resources of a personal computer. We applied our deep learning framework to map cancer-specific somatic mutation rates for 37 cancer types present in the Pan-Cancer Analysis of Whole Genomes (PCAWG) dataset^12^, using high-resolution epigenetic assays from healthy tissues as predictive features (well-known correlates of tumor mutation rates at the megabase scale^16,28^). We then used Dig to identify new coding and non-coding candidate cancer drivers in publicly available whole-genome, whole-exome and targeted sequencing cancer datasets. Our mutation maps are publicly available both as an interactive genome browser and as a standalone software tool for quantifying excess somatic mutations anywhere in the genome in a dataset of interest.

## Results

### Testing mutational excess with probabilistic deep learning

**Fig. 1:**
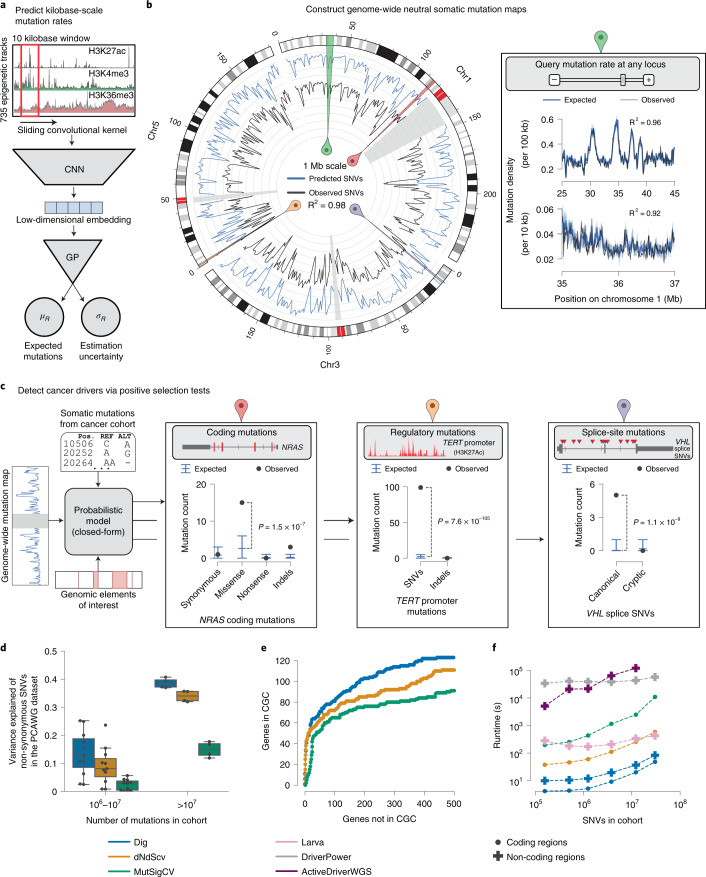
Modeling the genome-wide neutral somatic mutation rate and identifying cancer driver elements. **a**, Deep learning scheme to predict expected number of somatic mutations and prediction uncertainty using epigenetic sequencing of healthy tissue from the Roadmap Epigenomics consortium and ENCODE. **b**, Genome-wide neutral somatic SNV map and observed density of SNVs in 1-Mb windows from the PCAWG cohort (*n* = 2,279 samples). For clarity, only chromosomes 1, 3 and 5 are shown. Highlighted regions correspond to panels with the matching colored symbol. Inset: region on chromosome 1 modeled at 100-kb and 10-kb resolution. The reported R^2^ statistic between observed and expected SNV counts was calculated genome-wide. **c**, Examples of burden tests in the PCAWG dataset (*n* = 2,279 samples) for coding mutations in *NRAS* (*n* = expected versus observed mutations; synonymous: 0.81 versus 1; missense: 2.62 versus 15; nonsense: 0.22 versus 0; indels: 0.23 versus 3), non-coding mutations in the *TERT* promoter (SNVs: 2.12 versus 99; indels: 0.14 versus 0) and splice site SNVs in *VHL* (canonical splice SNVs: 0.03 versus 5; cryptic splice SNVs: 0.17 versus 0). Expected is mean with 95% CIs. *P* values from Dig. **d**, Proportion of variance of non-synonymous SNV count in genes 1–1.5 kb in length (*n* = 3,740 genes) in 16 PCAWG cohorts explained by different methods (size of each cohort reported in Supplementary Table 1). Box plot elements are defined in Methods. **e**, Approximate numbers of false-positive and true-positive driver genes identified in the PCAWG cohort by method (across a range of calling thresholds). Numbers are approximated because the true set of driver genes is unknown. CGC genes were used as a conservative approximation of true positives (a non-CGC gene may still be a true driver). **f**, Runtime of coding and non-coding driver detection methods. Comparison was restricted to SNVs because not all methods support indels. Coding analysis over *n* = 19,210 genes for Dig and dNdScv and *n* = 18,862 genes for MutSigCV. Non-coding analysis over *n* = 139,404 elements for Dig, DriverPower and Larva and *n* = 117,180 of those elements for ActiveDriverWGS. ActiveDriverWGS required >2 days to analyze the largest cohort.

**Extended Data Fig. 1:**
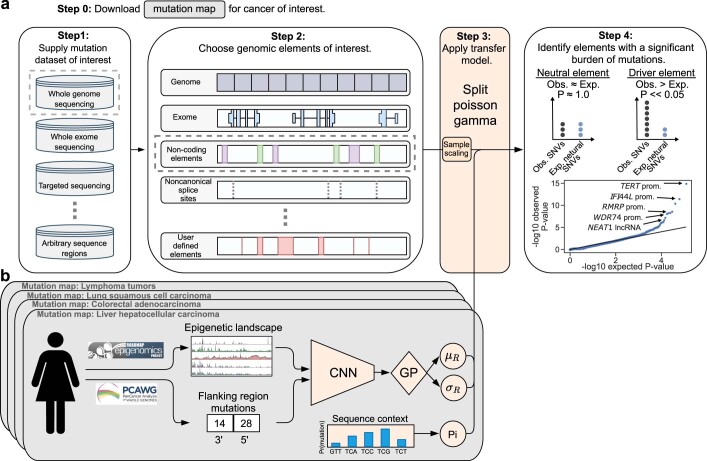
Detailed overview of the Dig model. **a**, Dig takes as input somatic mutations (SNVs and/or indels) (**Step 1**) identified from a cancer cohort sequenced with any methodology and a set of genomic elements of the user’s interest (**Step 2**). The neutral mutation rate from an available neutral somatic mutation map (detailed in panel **b**) is transferred to the selected SNV dataset via a closed-form probabilistic model (a split-Poisson gamma distribution^31^), that infers only a single scaling parameter at runtime (**Step 3**); then, a P-value for positive selection is calculated for each element by comparing the number of observed mutations to the number of expected neutral mutations (**Step 4**). **b**, A neutral mutation map for a particular cancer consists of 1) the mean and variance of the number of neutral mutations in kilobase-scale regions of the genome (default: 10 kb) as inferred by a convolutional neural network (CNN) and Gaussian process (GP) based on 735 epigenetic features from the Roadmap Epigenomics dataset and ENCODE (and optionally the number of mutations observed 100 kb up- and downstream of the region in the a training cancer cohort dataset); and 2) a sequence context model that provides the genome-wide likelihood of a mutation given its sequence context (default: trinucleotide sequences).

To enable rapid evaluation of mutational excess anywhere in the genome, we designed Dig to model somatic mutation rates genome-wide for a given type of cancer. Thus, the distribution of neutral mutations over any set of genomic positions for a cohort of tumors from that cancer type can be looked up nearly instantaneously. The method employs a probabilistic deep learning model that explicitly captures two central determinants of somatic mutation rate variability^16,17,21^: (1) kilobase-scale variation driven by epigenomic properties, such as replication timing and chromatin accessibility, that broadly impact efficacy of DNA repair^9^; and (2) base-pair-scale variation driven by the sequence context biases of processes that induce somatic mutations, such as APOBEC-driven cytidine deamination and UV light exposure^10,17,29,30^. Kilobase-scale variation is modeled with a custom deep learning architecture^31^ that uses a neural network to predict cancer-specific mutation rates within 10-kb regions and a Gaussian process (GP) to quantify the prediction uncertainty, taking as input high-resolution epigenetic assays (and, optionally, flanking mutation counts) (Fig. 1a, Extended Data Fig. 1 and Methods). By strictly partitioning the genome into non-overlapping train, validation and held-out test sets with five-fold cross-validation (predicting mutation rates in each one-fifth of the genome using a model trained and validated on observed mutations in the remaining four-fifths; Methods), the network constructs a kilobase-scale map of the mutation rate genome-wide for a given type of cancer (Fig. 1b). Base-pair variation is subsequently modeled using a generative graphical model that simulates how mutations should be distributed to individual positions in a region according to the nucleotide biases of mutational processes (Supplementary Fig. 1 and Methods). The marginal distribution over the number of neutral mutations at any set of positions has a closed-form solution that depends on the predicted regional mutation rate, the prediction uncertainty and the genome-wide probability that a position is mutated based on its neighboring nucleotides (Methods). Thus, once values for these parameters are learned from a training cohort of a given cancer type, the distribution of mutations expected at any set of positions in the genome can be queried for any tumor cohort of the same cancer and used to test for evidence of positive selection by quantifying if excess mutations are observed (Fig. 1c and Methods).

**Extended Data Fig. 2:**
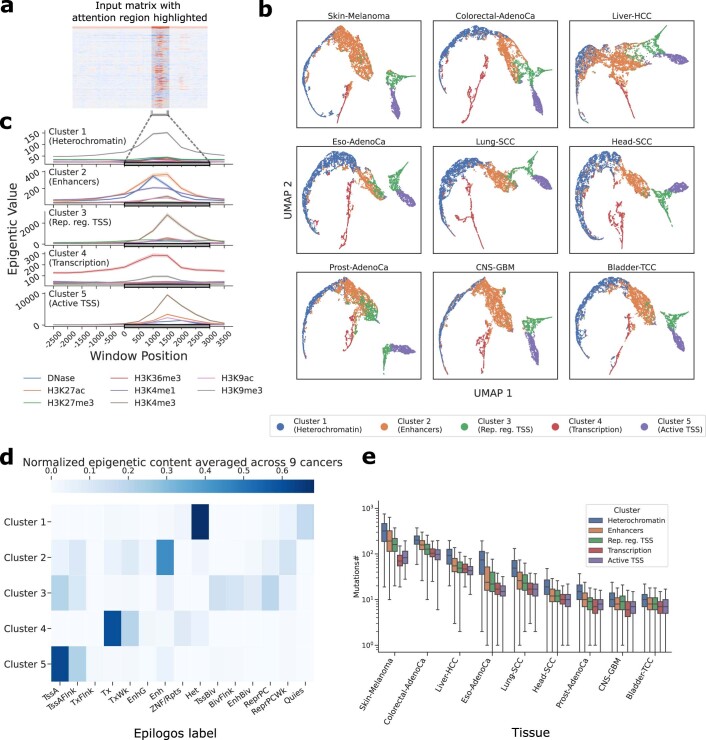
Epigenetic input features used by Dig to predict mutation density in nine cancer types. **a**, An example of a feature map across the 735 input features in a 50 kb region. The attention column is highlighted. **b**, UMAP visualization of the epigenetic content within attention columns, produced by averaging the same chromatin marks (for example, H3K27ac) across tissues, for nine types of cancer. The epigenetic content consistently formed five clusters in each cancer type. **c**, An example of the average epigenetic content of each cluster from lung squamous cell carcinoma. Each chromatin mark is the average across tissues with 95% CI. **d**, The epigenetic content of each cluster as determined by epilogos^74^, averaged across the nine cancer types. **e**, Boxplots of the number of mutations in regions containing an attention column from a given cluster, stratified by cancer type (boxplot elements defined in Methods). Skin-melanoma: N = 107 samples, Colorectal-AdenoCa: N = 50 samples, Liver-HCC: N = 314 samples, Eso-AdenoCa: N = 97 samples, Lung-SCC: N = 47 samples, Head-SCC: N = 56 samples, Prost-AdenoCa: N = 199 samples, CNS-GBM: N = 39 samples, Bladder-TCC: N = 23 samples.

We constructed mutation rate maps and inferred nucleotide mutation biases for 37 cancer types (Supplementary Tables 1 and 2 and Supplementary Data File 1) based on somatic mutations from the PCAWG dataset^12^ and 100-bp patterns of 723 chromatin marks in 111 tissues from Roadmap Epigenomics^32^, replication timing from ten cell lines from ENCODE^33^, and average nucleotide and GC content of the reference genome (Supplementary Table 3). We then benchmarked the accuracy of our somatic mutation rate models using the metric of proportion of variance explained, which we calculated as the square of the correlation coefficient between predicted and observed mutation counts as in previous work^16^. Dig successfully predicted a median of 77.3% (mean, 70.6%; range, 22.7–92.3%) of variance in observed single nucleotide variant (SNV) rates in 10-kb regions and a median of 94.6% (mean, 91.9%; range, 73.1–98.0%) of variance in 1-Mb regions (Fig. 1b, Supplementary Table 4 and Methods) across 16 cancer types for which benchmarking power was sufficient (>1 million mutations and excluding lymphomas, in which activation-induced cytidine deaminase produces extreme outlier mutation counts in locally hypermutated regions). Compared to existing methods designed specifically to analyze tiled regions^34^, coding sequence^4,21^ and non-coding elements in which synonymous mutations cannot be used to calibrate mutation rate models^18,19^ (for example, enhancers and non-coding RNAs), Dig explained the most variation of SNV counts within 10-kb regions in 14 of 16 cohorts, of non-synonymous SNV counts in 16 of 16 cohorts and of enhancer and non-coding RNA SNV counts in 15 of 16 cohorts, respectively (Fig. 1d, Table 1, Supplementary Fig. 2 and Supplementary Tables 4–6). Our approach’s accuracy is attributable, in part, to the ability of the deep learning network to identify local epigenetic structures, such as active transcription start sites, and to associate these structures with mutation rates (Extended Data Fig. 2 and Supplementary Note 1).

**Table 1:** Proportion of variance in observed SNV counts in the PCAWG cohort (*n* = 2,279 samples) explained by different methods

	Percent of variance explained in observed SNV count (Pearson R^2^ between observed and predicted SNV counts)		
Method	10-kb regions	Non-synonymous SNVs in coding sequences	Enhancers and non-coding RNAs
Dig (this work)	**92.3%**	**39.5%**	**49.0%**
NBR^34^	85.3%		
dNdScv^4^		35.7%	
MutSigCV^21^		17.8%	
Larva^18^			26.4%
DriverPower^19^			47.5%

To minimize confounding from variation in element length (as longer elements are expected to have more mutations on average than shorter elements), the comparisons were restricted to genes with coding sequence 1–1.5 kb in length (*n* = 3,740 genes) and to non-coding elements 0.5–1 kb in length (*n* = 7,412 elements). A blank entry indicates that the method did not produce predictions over the associated annotation (NBR was able to analyze a subset of 6,024 enhancers and non-coding RNAs; it explained 1.8% of SNV count variation in those regions).

This accuracy enabled correspondingly powerful driver identification. In benchmarks testing downstream ability to identify evidence of positive selection (that is, excess of mutations) within previously identified driver elements, Dig matched or exceeded the performance of methods tailored toward specific classes of elements^4,18–21^ in whole-genome and whole-exome sequenced samples (Fig. 1e, Supplementary Figs. 3–5, Supplementary Tables 7–10 and Supplementary Notes 2 and 3). Considering driver genes—for which high-quality databases of known driver genes that can approximate gold standard true positives exist (Methods)—Dig had the highest F1-score (a measure of accuracy) in 24 of 32 PCAWG cohorts (excluding skin and blood cancers as in previous work^19^ due to local hypermutation processes) and the most power in 14 of 16 whole-exome cohorts compared to widely used, burden-based driver gene detection methods (Fig. 1e, Supplementary Figs. 3 and 4 and Supplementary Tables 8 and 9) (power was measured as the area under approximated receiver operating characteristic curves, which could be estimated due to the larger sizes of the exome-sequenced cohorts; Methods).

Identifying potential driver elements with Dig was 1–5 orders of magnitude faster than existing methods that train new models for every element and cohort analyzed (Fig. 1f). For example, testing 10^7^ observed mutations for evidence of positive selection within 10^5^ non-coding elements with Dig completed in <90 seconds on a single CPU core compared to between ~10 minutes and >2 days for other methods. Thus, our method matches or exceeds the power of existing approaches while requiring less runtime and providing flexibility to identify drivers with mutation-level precision genome-wide.

### Small mutation sets increase power to identify drivers

Previous searches for non-coding driver elements have concluded that such drivers are likely rare, carried by <1% of samples^5^. A power analysis using our model’s generative capabilities concurred (Methods), indicating the most known non-coding elements (for example, enhancers) require at least 1–2% of samples to carry driver mutations to have a >90% likelihood of detecting mutational excess at current sample sizes (~10^2^ for individual cancer types; ~10^3^ for pan-cancer cohorts) (Supplementary Fig. 6). However, by reducing the size of tested elements to encompass only tens to hundreds of positions (as opposed to the thousands of base pairs spanned by most non-coding elements considered to date—for example, average enhancer size: 1,717 bp; range, 600–30,200 bp), power to identify driver mutations in <1% of samples increased by ~20% (Supplementary Fig. 6). To demonstrate the ability of Dig to find putative drivers, we, thus, defined and tested specific sets of mutations with potential functional impact for evidence of selection. The ability to test user-specified sets of specific mutations genome-wide is a unique feature (to our knowledge) of our method.

### Quantifying pan-cancer selection on cryptic splice SNVs

**Fig. 2:**
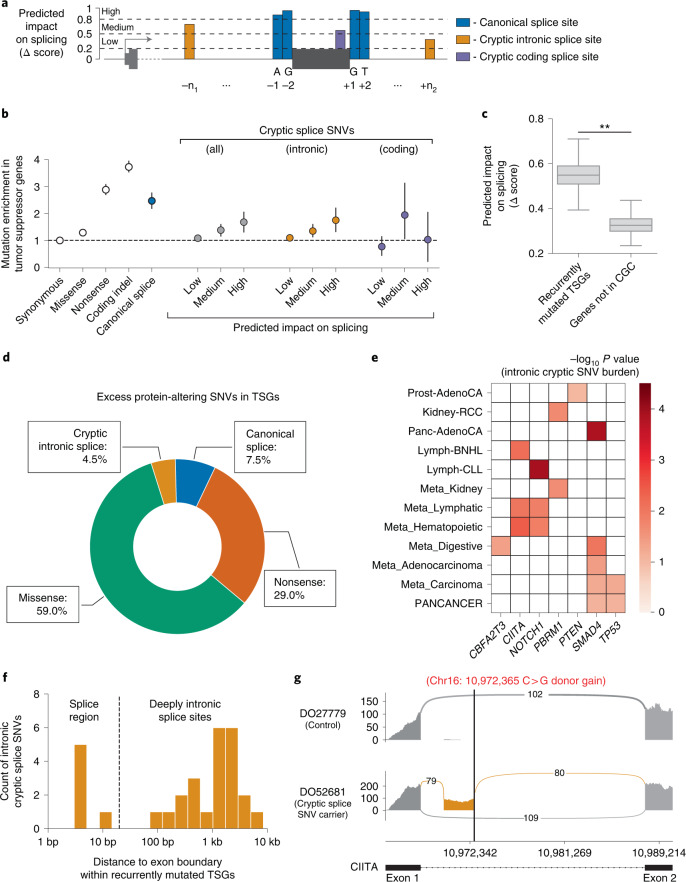
Evidence of positive selection on intronic cryptic splice SNVs in TSGs. **a**, Schematic of the splice-altering SNVs considered in this analysis. Predicted impact on splicing measured by the SpliceAI Δ score (higher score approximates higher likelihood of altered splicing). We stratified possible SNVs by predicted impact on splicing: low predicted impact (0.2 < Δ < 0.5), medium predicted impact (0.5 < Δ < 0.8) and high predicted impact (0.8 < Δ < 0.1). **b**, Estimated enrichment (with 95% CI) of observed mutations compared to expected neutral mutations in TSGs stratified by variant type and predicted impact on splicing in *n* = 2,279 pan-cancer samples from the PCAWG dataset (*n* mutations per category in Supplementary Table 11). **c**, Predicted splicing impact (SpliceAI Δ score) for intronic cryptic splice SNVs observed in recurrently mutated TSGs (see **e**) compared to those observed in genes not in the CGC (** indicates bootstrapped *P* < 3 × 10^−4^; Methods). Box plot elements are defined in Methods. **d**, Proportion of excess SNVs in TSGs contributed by each protein-altering SNV category. **e**, Known TSGs per cancer with a significant burden (FDR < 0.1) of predicted intronic cryptic splice SNVs (*n* mutations per gene in Supplementary Table 13). **f**, Distribution of distance to nearest exon boundary for the intronic cryptic splice SNVs observed in recurrently mutated TSGs. **g**, Pileup of RNA-seq reads in a Lymph-BNHL carrier of a predicted, deeply intronic cryptic splice SNV (labeled in red) in *CIITA* and a control Lymph-BNHL sample, showing the inclusion of a cryptic exon (gold) in the cryptic splice SNV carrier. Arc labels indicate the number of RNA-seq reads that support each exon junction.

**Extended Data Fig. 3:**
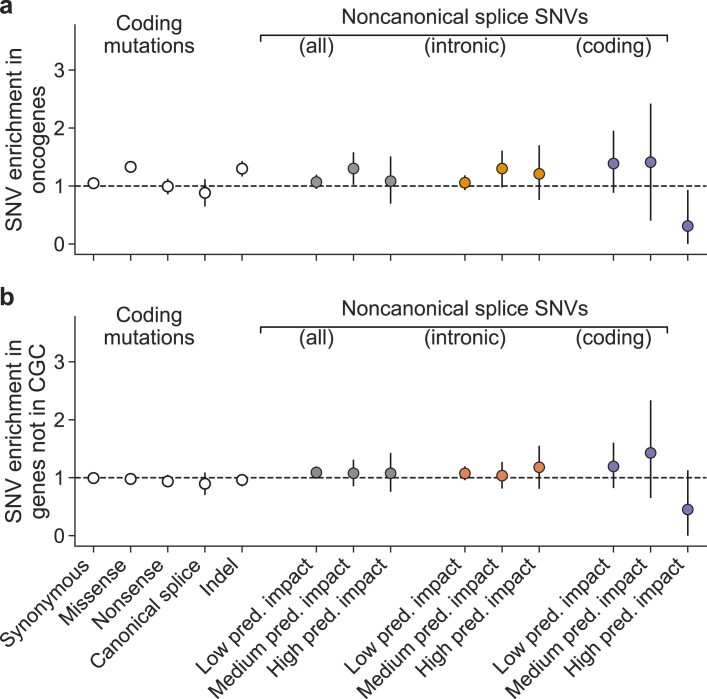
Cryptic splice SNV enrichment in oncogenes and genes not in the CGC. Estimated SNV enrichment with 95% CIs as in Fig. 3b for oncogenes in the CGC, **a**, and 500 randomly selected genes not in the CGC, **b**. Enrichment is not significant in any category after accounting for multiple hypothesis testing except missense mutations and indels in oncogenes, as expected. (N = 2,279 samples in each panel; number of mutations per category in Supplementary Table 11).

Alternative splicing is increasingly recognized as functionally relevant to cancer^35,36^, and recent studies have associated specific somatic mutations outside canonical splice sites with alternative splicing events observed in expression data^37,38^. We, thus, applied Dig to rigorously quantify the extent to which cryptic splice SNVs, which may exist in both exons and introns of a gene (Fig. 2a), occur in excess of the neutral mutation rate and, therefore, may function as driver mutations under selection. In tumor suppressor genes (TSGs) from the Cancer Gene Census (CGC)^39^, cryptic splice SNVs as predicted by spliceAI^40^ (Methods) occurred significantly more often than expected under neutrality (648 SNVs observed in 283 TSGs versus 550 SNVs expected; *P* = 2.38 × 10^−5^) (Fig. 2b and Supplementary Tables 11 and 12); were primarily enriched in introns (where most such mutations occur); and were biased to occur in sites with high predicted impact on splicing (SNVs with predicted impact Δ score >0.8 exhibited a 1.75-fold enrichment (95% confidence interval (CI): 1.31–2.22 fold), *P* = 2.52 × 10^−5^) (Fig. 2b,c). Overall, intronic cryptic splice SNVs were estimated to account for 4.5% (95% CI: 1.3–7.4%) of excess (potential driver) SNVs in TSGs, similar in magnitude to the 7.4% (5.6–9.7%) attributable to canonical splice SNVs, whose driver potential is well-established^4^ (Fig. 2d) (exonic excess SNV estimates were consistent with estimates from dNdScv; Supplementary Fig. 7). Results were robust to high mutation burden samples (Supplementary Fig. 8) and consistent with an analysis that did not rely on our mutation maps (Supplementary Fig. 9). Neither control genes not in the CGC nor oncogenes in the CGC were enriched for cryptic splice SNVs (Extended Data Fig. 3 and Supplementary Table 11). The lack of enrichment in oncogenes suggests that gain-of-function splice mutations beyond those that induce skipping of *MET* exon 14 are extremely rare, which may reflect the low likelihood of an intronic splice mutation resulting in the in-frame addition of residues that pathologically activate an oncogene. Conversely, the enrichment in TSGs suggests that cryptic splice mutations are generally inactivating, likely by triggering nonsense-mediated decay of mRNA transcripts or generating a protein with impaired function.

Considering individual genes, seven TSGs in 12 cancer types had a significant burden of intronic cryptic splice SNVs (false discovery rate (FDR) < 0.1 for *n* = 283 TSGs in 37 cancers) (Methods, Fig. 2e and Supplementary Table 13), with patterns of TSG–cancer associations consistent with known tissue specificity of TSGs. Pan-cancer, *TP53* and *SMAD4*—both implicated in many cancers—carried an excess of cryptic splice SNVs. In contrast, the hematopoietic-specific TSG *CIITA* and the renal-specific TSG *PBRM1* carried excess cryptic splice SNVs in blood and kidney malignancies, respectively. In further support of these associations, the intronic cryptic splice SNVs observed in these TSGs, most (79.3%) of which fell outside annotated splice regions (that is, >20 bp from exon–intron boundaries) (Fig. 2f), had significantly higher predicted impact on splicing than those observed in genes not in the CGC (Fig. 2c) (mean SpliceAI Δ score = 0.55 versus 0.33; *P* < 3 × 10^−4^; Methods). Moreover, of the six cryptic splice SNV carriers with available RNA sequencing (RNA-seq) data with sufficient coverage, five had evidence of alternative splicing (Fig. 2g, Supplementary Fig. 10, Supplementary Table 14 and Supplementary Note 4) as quantified by LeafCutter^41^ (Methods). Overall, these results provide evidence that intronic cryptic splice SNVs are under positive selection in TSGs and likely act as driver events in several percent of tumors across multiple cancer types.

Nine genes not in the CGC also had a significant burden of intronic cryptic splice SNVs in six cancers (Supplementary Table 15) at FDR < 0.1, of which two genes had a significant burden at the more stringent Bonferroni (α < 0.05) correction for 712,600 tests conducted across all genes and cancers. The burdens of four genes were driven by recurrent mutations at a single intronic location per gene (Supplementary Table 16). Implicated genes include *BTG2* in lymphoma, which is involved in the regulation of the G1/S transition of the cell cycle and has recently been implicated as a driver of blood cancers based on mutations in its coding sequence^10^, and *ADAM19* in hemopoietic tumors, which has been implicated in the oncogenesis of breast^42^, prostate^43^, colorectal^44^ and ovarian^45^ cancers. Although the computational prediction of new drivers should be interpreted with caution (Discussion), these genes may be promising targets for future experimental studies to investigate their potential tumorigenic properties.

### Non-coding candidate cancer driver mutations in 5′ untranslated regions

**Fig. 3:**
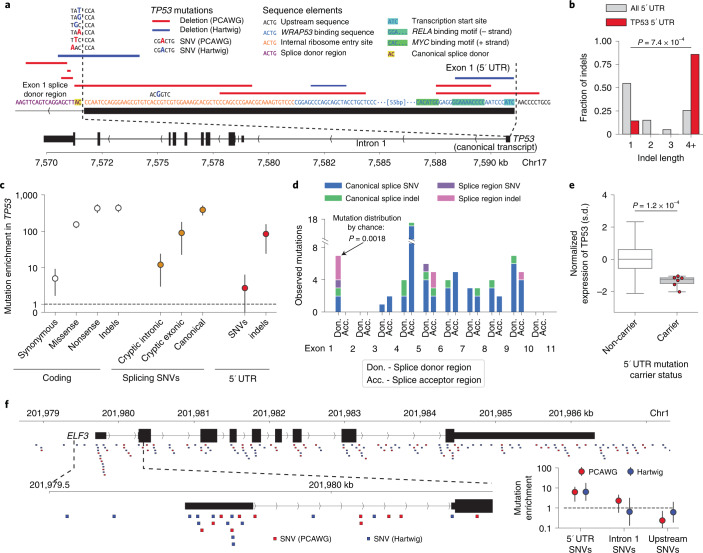
Enrichment of somatic mutations in the 5′ UTRs of TP53 and ELF3. **a**, Mutations from the PCAWG and Hartwig Medical Foundation cohorts observed within exon 1 of the 5′ UTR of the canonical TP53 transcript. DNA sequence from GRCh37 reference genome (+ strand). Mutation types, relevant sequence and regulatory elements as indicated in the legend. **b**–**e**, Analysis on PCAWG dataset (*n* = 2,279 samples). **b**, Distribution of indel sizes observed within 5′ UTRs of genes other than TP53 (*n* = 3,988 indels) and within the TP53 5′ UTR (*n* = 7 indels). *P* value comparing median indel lengths from one-sided Mann–Whitney *U*-test. **c**, Estimated mutation enrichment relative to the neutral mutation rate (observed / expected neutral mutations) within TP53 stratified by mutation type and location (number of mutations per category in Supplementary Table 17). Error bars, 95% CI. **d**, Distribution of mutations observed within donor and acceptor splice regions (defined as the 20 bp 3′ and 5′ of an exon, respectively) of the canonical TP53 transcript. Canonical splice SNVs and indels: mutations altering the two base pairs immediately adjacent to an exon boundary; splice region SNVs and indels: mutations intersecting the splice region but not the canonical splice sites. The donor splice region of exon 1 of the 5′ UTR (shown in **a**) is bolded. *P* value of observing the distribution of canonical and splice region mutations in the donor splice region of exon 1 5′ UTR compared to all other TP53 splice regions computed via a two-sided Fisher’s exact test. **e**, Expression of TP53 on standard deviation scale in carriers of TP53 5′ UTR mutations (*n* = 6) and non-carriers (*n* = 1,205), adjusted for tumor type and copy number in the PCAWG dataset (*n* = 2,279 samples). *P* value via one-sided Mann–Whitney *U*-test on adjusted and standardized expression values. Box plot elements are defined in Methods. **f**, SNVs overlapping ELF3 in the PCAWG and Hartwig Medical Foundation cohorts. Insets: zoom-in of the ELF3 5′ UTR region and estimated mutational enrichments with 95% CIs within this region (number of mutations per category in Supplementary Tables 17 and 18).

Hypothesizing that indels could have large effect size on gene expression by disrupting transcription factor binding motifs, we searched promoters (*n* = 19,251) for a burden of indels in the PCAWG dataset (Methods). The *TP53* promoter was the only element with a genome-wide significant (FDR < 0.1) burden of indels (7 observed versus 0.54 expected; *P* = 9.4 × 10^−7^) (Fig. 3a), consistent with a previous analysis that used restricted hypothesis testing to boost statistical power^5^. The observed mutations—all deletions significantly larger than expected (Fig. 3b) (median length = 17 bp versus 1 bp expected; *P* = 7.4 × 10^−4^, one-sided Mann–Whitney *U*-test)—specifically affected exon 1 of the canonical 5′ untranslated region (UTR), disrupted critical sequence elements (transcription start site, *WRAP53* binding sequence^46^, internal ribosome entry site^47,48^ and the donor splice region of the multi-exonic 5′ UTR) (Fig. 3a) and exhibited enrichment comparable to cryptic exonic splice SNVs in *TP53*, which are well-characterized cancer drivers^49^ (Fig. 3c). More than half of the mutations (four of seven) within the exon 1 splice region did not alter the canonical splice sites, an unexpected pattern compared to other *TP53* splice regions (Fig. 3d) (*P* = 1.8 × 10^−3^, two-sided Fisher’s exact test). The 5′ UTR mutation carriers had significantly lower expression of *TP53* than individuals without *TP53* mutations and individuals with predicted functional coding *TP53* mutations (1–2 standard deviation decreases in *TP53* expression compared to non-carriers, *P* = 1.2 × 10^−4^; Methods, Fig. 3e and Supplementary Fig. 11), suggesting that these mutations either directly inhibit *TP53* transcription or result in nonsense-mediated decay of the mRNA transcripts. Corroborating these results, seven of 2,399 distinct samples from the Hartwig Medical Foundation^50^ showed a similar mutational pattern, with three carrying >10-bp deletions and four carrying SNVs in *TP53* exon 1 and its donor splice region (Fig. 3a).

These results motivated a targeted search for mutational burden in 5′ UTRs and their splicing regions across 106 TSGs and 95 oncogenes with multi-exonic 5′ UTRs (Methods). One additional element, the 5′ UTR of *ELF3*, had a significant burden of SNVs (Fig. 3f) in PCAWG samples (6 observed SNVs versus 0.96 expected; *P* = 2.9 × 10^−4^); samples from the Hartwig Medical Foundation displayed a similar enrichment (10 observed versus 1.5 expected; *P* = 3.8 × 10^−4^; Methods). In both sets of samples, the enrichment was concentrated within the canonical *ELF3* 5′ UTR; surrounding sequences (upstream promoter and intron 1) were not enriched for mutations (Fig. 3f). The 16 mutations largely altered distinct base pairs within the 5′ UTR—although two positions mutated in PCAWG samples were also mutated in the Hartwig samples—suggesting that this 5′ UTR might be broadly sensitive to perturbation, possibly by prompting changes in promoter methylation that alter *ELF3* expression^51^. An alternative possibility could be an unmodeled local mutational process or technical artifact in this region^9^; however, a careful analysis did not find evidence for any such features that have explained other non-coding mutational hotspots^5^ (Supplementary Note 5). The small number of carriers and limited availability of transcriptomic assays (only three carriers from PCAWG had RNA-seq data) prevented investigation into the possible function of these 5′ UTR mutations. Thus, additional follow-up, particularly experimental assays assessing the impact of 5′ UTR mutations^52^, will be necessary to determine whether the mutational enrichment here represents positive selection or represents a new neutral mutational process.

### The shared landscape of common and rare driver genes

Small sample sizes have limited assessment of whether rare coding mutations (which account for most exonic mutations in tumors) act as drivers even in well-characterized driver genes. We increased statistical power in two ways: (1) by analyzing large meta-cohorts of non-synonymous SNVs from 14,018 whole-exome and targeted sequencing samples, representing ten solid tumor types (median samples per cancer, 1,195; range, 515–3,110) (Supplementary Table 19 and Methods); and (2) by considering only activating mutations in oncogenes (obtained from the Cancer Genome Interpreter^23^) and predicted loss-of-function (pLoF) mutations in all other genes. Such analysis has previously been impeded by the exclusion of synonymous mutations from large, publicly available targeted sequencing datasets^53–57^ because existing driver gene detection methods are reliant upon synonymous mutations. Dig circumvents this difficulty because model parameters have already been inferred from a separate training cohort.

**Fig. 4:**
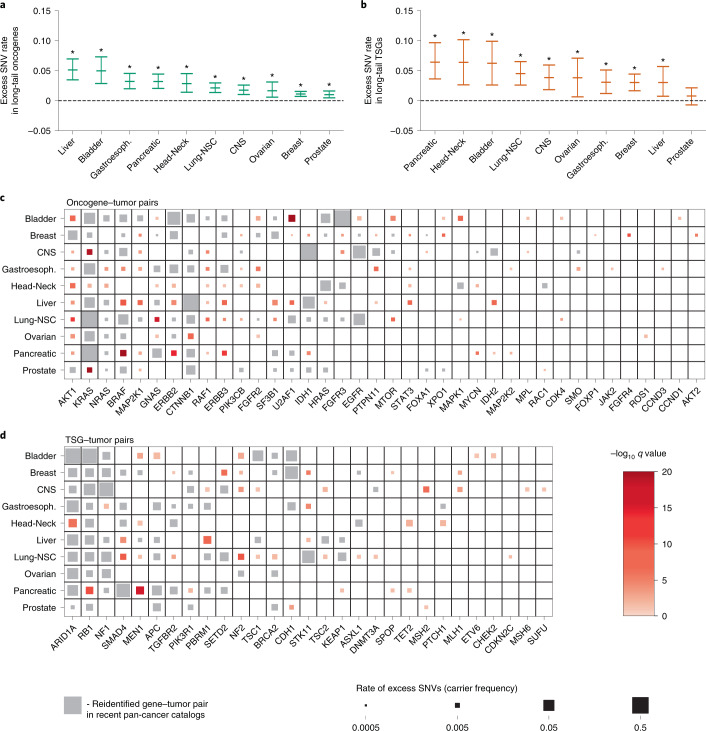
Enrichment of protein-altering SNVs in ‘long-tail’ genes reveal a shared landscape of common and rare driver genes. **a**,**b**, Estimated mutation rates with 95% CIs of excess oncogenic SNVs in oncogenes (**a**) and pLoF variants in TSGs (**b**) that were not previously associated with a given cancer (*x* axis) in three large driver gene catalogs^7,10,11^. Stars indicate that the burden of oncogenic (pLoF) SNVs was significant in long-tail oncogenes (TSGs) in the cancer type (*P* values and number of SNVs per category are in Supplementary Tables 20 and 21). **c**,**d**, Oncogene–tumor pairs and TSG–tumor pairs with a significant burden of oncogenic or protein-truncating SNVs. Gene–tumor pairs previously reported by Dietlein et al.^10^, Bailey et al.^11^ or Martínez-Jiménez et al.^7^ are marked in gray. Pairs that are not present in those catalogs are marked in red, with color intensity indicating significance of association. Marker size is proportional to the estimated rate of excess mutations after accounting for cancer-specific neutral mutation rates. CNS, central nervous system; NSC, non-small-cell.

**Extended Data Fig. 4:**
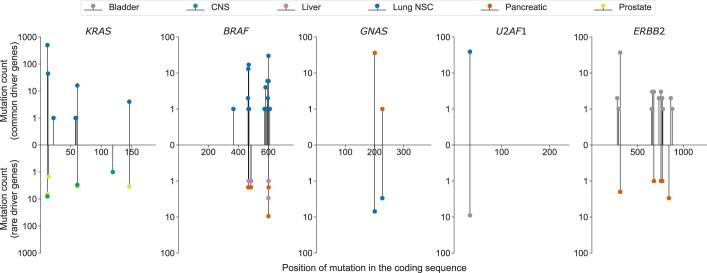
Examples of distribution of activating mutations in gene-tumor pairs. Top y-axis: distribution in cancers for which the gene is a known common driver. Bottom y-axis: distribution in cancers for which the gene is a newly proposed rare driver. The genes shown are the five long-tail genes with the highest carrier frequency across the cancer types tested. Color of the ball indicates cancer type.

For each cancer, we first restricted our analysis to ‘long-tail’ genes, which we defined as oncogenes and TSGs not associated with that cancer type in any of three recent, large, pan-cancer surveys of driver genes^7,10,11^. Dig estimated that 1–5% of samples (depending on the cancer) carried activating SNVs in long-tail oncogenes (Fig. 4a) and 3–6.5% carried pLoF SNVs in long-tail TSGs (Fig. 4b). These rates were significantly higher than expected (*P* < 3.78 × 10^−9^ for activating SNVs in all cohorts; *P* < 3.10 × 10^−4^ for pLoF SNVs in all cohorts except prostate (*P* = 0.056 for prostate)) (Supplementary Fig. 12, Supplementary Tables 20 and 21 and Methods). These rates were consistent when we restricted the analysis to only whole-exome sequenced samples, although power to detect positive selection was decreased due to reduced sample size (Supplementary Fig. 13 and Supplementary Tables 22 and 23). Considering individual genes, 92 oncogene–tumor pairs not reported in recent pan-cancer surveys of driver genes had a significant (FDR < 0.1) burden of activating SNVs (Fig. 4c and Supplementary Table 24). Forty-six TSG–tumor pairs not reported in the pan-cancer surveys had a significant burden of pLoF mutations (Fig. 4d and Supplementary Table 25). The newly identified candidate driver genes were rare compared to driver genes in existing databases (0.28% (interquartile range, 0.14–0.53%) versus 1.3% (interquartile range, 0.59–3.0%) for newly implicated and known driver genes, respectively; *P* = 3.1 × 10^−27^, two-sided Mann–Whitney *U*-test). Further supporting these predictions, the distribution of activating mutations in a given driver gene was similar between cancers in which the gene is a known, common driver and cancers in which we newly implicated the gene as a putative rare driver (Extended Data Fig. 4). For example, the G12, G13, Q61 and A146 positions of *KRAS* accounted for most *KRAS* SNVs in both common and rare scenarios (lung non-small-cell tumors: 568/586 mutations; prostate tumors: 12/17 mutations; gliomas: 11/15), and the V600E mutation accounted for the plurality of *BRAF* SNVs in common and rare scenarios despite each gene having dozens of known activating SNVs (52 and 71, respectively). Additionally, carriers of mutations in several predicted rare driver genes exhibited phenotypes consistent with those reported in tumors in which the genes are common drivers (Supplementary Note 6). For example, central nervous system tumors with rare pLoF mutations in the DNA mismatch repair genes *MSH2* and *MLH1* exhibited significantly increased global mutation rates across 213 targeted sequenced genes (*MSH2*: mean 30.1 mutations in carriers versus 3.0 in non-carriers; *P* = 3.8×10^−7^, one-sided Mann–Whitney *U*-test; *MLH1*: mean 35.3 mutations in carriers versus 3.1 in non-carriers; *P* = 8.8×10^−6^, one-sided Mann–Whitney *U*-test).

A further 29 gene–tumor pairs had a significant (FDR < 0.1) burden of pLoF mutations in genes not in the cancer driver databases for any cancer (Methods and Supplementary Table 26), of which two were significant at the more stringent Bonferroni (α < 0.05) correction for the total number of genes tested, and six were additionally supported by a nominal (*P* < 0.05) burden of missense mutations. The top hit is the cell polarity gene *PARD3* in gastroesophageal cancer (9 observed pLoF SNVs versus 1.1 expected; *P* = 1.57 × 10^−6^), which, despite not appearing in major driver gene databases, is a known fusion partner of the oncogene *RET* and has been implicated in the tumorigenesis of multiple solid cancers^58^. The ability to distinguish mutational burdens in genes with a low frequency of mutations, such as *PARD3* (nine carriers in 827 samples), highlights the increased statistical power that our approach can achieve by testing specific sets of mutations in large cohorts for evidence of positive selection.

Our results represent progress toward an unbiased, pan-cancer catalog of driver genes and suggest that driver mechanisms are shared across the common and rare driver landscape of solid cancers. However, computational identification of rare driver genes at current sample sizes relies upon small mutation counts, and predictions should be interpreted with care. Experimental characterization of the functions of genes in the relevant cancers is essential to confirming their carcinogenic roles.

## Discussion

Dig is a probabilistic deep learning method that enables rapid tests for evidence of positive selection on genomic elements that can be defined with the precision of individual mutations anywhere in the genome. The strong performance of the method in modeling mutation rates and identifying candidate drivers highlights the power of deep learning to capture complex cellular processes with data derived from high-throughput sequencing^40,59–63^. Specifically, building upon the observation that epigenetics correlate with somatic mutation rates^17^, we showed that neural networks applied to a corpus of high-resolution chromatin immunoprecipitation followed by sequencing (ChIP-seq) assays are able to learn nuanced, non-linear associations between local epigenetic structures and patterns of somatic mutations. Moreover, techniques presented here are adaptable to other contexts. For example, quantification of prediction uncertainty by coupling a Gaussian process to the final layer of a neural network may be a practical solution to improve the reliability and interpretability of predictions in other deep learning settings^64^.

The application of our high-resolution mutation rate maps to quantify mutational burdens genome-wide provides a glimpse into the landscape of rare and non-coding driver mutations that we anticipate will emerge as cancer sequence sample sizes continue to grow. Although the driver candidates we report—in cryptic splice sites, 5′ UTRs and rarely mutated genes—occurred at low frequencies individually, our estimates suggest that they collectively contribute to the disease pathology of up to 10% of tumors (summing across the percent of tumors predicted to carry excess mutations in each of these elements). This estimate may be conservative, as several analyses used datasets of mutations that are unlikely to be comprehensive (for example, catalogs of predicted cryptic splice SNVs and known activating SNVs). The quantification of these rare driver events is important, in part, because it suggests avenues to expand patient treatment options by repurposing therapeutics; a targeted therapy approved for a mutation in one cancer type may prove beneficial to patients with the same mutation in other cancer types. Indeed, cancer-agnostic approaches to patient stratification are currently being deployed at some cancer centers^65^.

Additionally, current sample sizes are not adequate to uncover infrequent drivers under moderate or weak positive selection. We anticipate that Dig will be particularly useful in uncovering such mutations due to its ability to rapidly evaluate mutations spread over large swaths of the genome. For instance, a preliminary analysis that we performed on enhancer networks identified several genes with a burden of enhancer mutations (Supplementary Table 27 and Supplementary Note 7), including *FOXA1*, in which promoter mutations are thought to drive breast cancer by increasing gene expression^66^. A possible approach to increase sample size with existing data is to call somatic mutations in regions flanking coding sequence using off-target reads from large targeted or whole-exome sequenced clinical cohorts.

However, computational prediction alone is not sufficient to establish the causal role of an element or mutation in cancer pathology because an excess of mutations compared to the neutral mutation rate does not definitively prove positive selection. Moreover, recent studies have shown that canonical cancer driver mutations can be present in seemingly healthy tissues^67–71^, adding an additional layer of complexity to interpreting whether or how a mutation causally contributes to a malignant phenotype. Ultimately, experimental validation is necessary to establish the causal role for a mutation as a driver of cancer. Dig provides a tool for in silico guidance of in vitro and invivo studies because it enables prioritization of precise sets of mutations that may act as drivers in both the coding and non-coding genome. These specific sets of mutations can then be evaluated in experimental systems. For example, the predicted cryptic splice mutations that Dig identified as putative drivers could be evaluated as possible drug targets by CRISPR base editing of cell lines, followed by drug screening assays^72^. Thus, we anticipate that deep learning generally, and our tool specifically, can improve computational, experimental and clinical utility of the growing body of cancer genome sequencing data.

## Methods

### Sequencing data curation

#### PCAWG dataset

We obtained somatic SNVs and indels from whole-genome sequencing of 2,583 unique tumors from the International Cancer Genome Consortium (ICGC) data portal (https://dcc.icgc.org/) and the database of Genotypes and Phenotypes (dbGaP) (project code: phs000178) that previously passed quality control^5^. The somatic mutation calls in this dataset have previously been stringently filtered to remove possible germline calls, false-positive calls due to oxidative DNA damage and calls with high strand bias^12^. Following procedures described in Rheinbay et al.^5^, we grouped samples into 38 individual cancer types and 14 meta-cohorts that combined similar tumor types, including a pan-cancer cohort that included all samples except melanoma and lymphoma tumors (consistent with Rheinbay et al.^5^). We removed samples with reported high microsatellite instability from all cohorts except the pan-cancer cohort and annotated autosomal coding SNVs and indels with their predicted functional impact using a custom annotation method. (We excluded sex chromosomes because the number of observed mutations on the X chromosome depends on the sex composition of a cohort). For the creation of somatic mutation maps and driver element analysis, we considered cohorts with at least 20 samples and >10^5^ SNVs (Supplementary Table 1). This resulted in a set of 23 individual cancer types and 14 meta-cohorts.

#### Dietlein et al. dataset

We obtained somatic SNVs and indels from whole-exome sequencing of 11,873 tumors from 28 cancer types that had previously been curated in Dietlein et al.^10^ from http://www.cancer-genes.org/; the dataset previously underwent filtering to remove germline calls and due to oxidative DNA damage, as described in Dietlein et al^10^. We restricted to a set of 8,617 tumor samples from 17 cancer types for which we had mutation rate models trained on the PCAWG dataset (Supplementary Table 28). We additionally constructed a pan-cancer dataset by merging somatic mutations from all samples excluding melanoma and hematopoietic malignancies as in PCAWG^5^. Coding mutations were annotated for their predicted functional impact as above.

#### Target sequencing datasets

We obtained somatic SNVs from targeted sequencing of ten types of solid cancers performed using the IMPACT protocol at Memorial Sloan Kettering Cancer Institute from cbioportal^53^ (https://www.cbioportal.org/) (Supplementary Table 19). Possible germline calls were previously excluded from these datasets. We removed duplicate patients and hypermutated samples with >100 coding mutations in 221 genes common to all whole-exome and targeted sequenced samples (removal of hypermutated samples is common in driver gene detection and has been shown to improve accuracy^4^). Coding SNVs were then annotated for their predicted functional impact in coding sequence as above and merged with SNVs from the whole-exome datasets (after removing hypermutated samples) of the corresponding cancer type to form mega-cohorts with aggregate sample size of 14,018 tumors in ten cancer types.

#### Additional filtering of germline mutations

Any mutation occurring in an element with a nominally FDR < 0.1 significant burden of mutations was cross-referenced with the Genome Aggregation Database (gnomAD) version 2.1.1 (ref. ^73^) and excluded if it occurred in gnomAD with an allele count of five or more in any population, unless the mutation occurred primarily in a single population and the carrier was not of that population (this occurred only once; the mutation 1:43804317-C>T was observed in a carrier of European ancestry but is reported in gnomAD as occurring in Latino/admixed American populations). If the mutational burden of the element did not remain FDR < 0.1 significant after exclusion of these possible germline mutations, it was removed from further analysis. This filter was applied to all datasets.

### Identification of mutational excess with probabilistic deep learning

Dig consists of two components: (1) a deep learning module that models approximately constant somatic mutation rates within kilobase-scale regions (for example, 10–50 kb) due to epigenetic features (for example, chromatin compactness) that vary at this scale^5^; and (2) a generative probabilistic model that captures the likelihood that a given position is mutated in a cancer cohort, conditioned on its sequence context^10,29,30,34^ and the kilobase-scale mutation rate of that cancer type. Intuitively, the kilobase-scale model provides information about how many neutral mutations should be present in a region, whereas the nucleotide context model determines how those mutations should be distributed among individual positions.

#### Modeling kilobase-scale mutation rates with deep learning

##### Model architecture

The purpose of the deep learning model is to (1) predict the mutation rate *μ*_*R*_ and (2) quantify prediction uncertainty $\sigma _R^2$ conditioned on the epigenetic organization of the region *R*. The architecture was previously described^31^. In brief, the network consists of a convolutional neural network (CNN) that takes as input a high-dimensional matrix of epigenetic assays (see ‘Model input and output’ section) and projects the matrix into a 16-dimensional vector. Optionally, the CNN also embeds into the 16-dimensional vector the mutation counts observed in the 100-kb regions flanking the region of interest. The low-dimensional embedding is then provided as input to a GP that predicts the mean and variance of number of mutations in the region. Technical details are provided in Supplementary Methods.

##### Model input and output

The CNN and GP were trained sequentially to predict somatic SNV counts in non-overlapping 10-kb regions by minimizing mean squared error loss between predicted values and observed counts from the PCAWG dataset for each of 37 cancer types. The network received as input matrices of size 735 × 100 where each row was an epigenetic feature track, and each column was the average track value in non-overlapping 100-bp windows. In total, 723 rows were uniformly processed −log_10_ *P* values for peaks of chromatin markers from 111 tissues; ten rows were replication timings of ten cell lines from ENCODE^33^; and two were the average nucleotide content and average GC content of the human reference genome (Supplementary Table 3). The network additionally received as input somatic SNV counts in 100-kb regions flanking each 10 kb of interest from the relevant cancer in the PCAWG dataset. However, the accuracy of the method over 1-Mb regions was benchmarked using networks trained without flanking region counts to avoid any leakage of information between train and test sets.

##### Model training

For each cancer, predictions in each non-overlapping 10-kb region *R* of the autosome was obtained via the following five-fold cross-validation strategy. Bins that passed quality control (Supplementary Methods) were randomly divided into five equal-size folds, each containing 20% of the bins. Sequentially, each fold was withheld, and a deep learning model was trained using 80% of the remaining bins and validated over the other 20% of the remaining bins to avoid overfitting (Supplementary Methods). Prediction was then performed over the held-out fold (20% of the genome) and over regions filtered by quality checks. Additional technical details of model training are described in Supplementary Methods.

#### Testing mutational burden with a graphical model

##### Genome-wide likelihood of mutation from sequence context

For each cancer, maximum likelihood estimation was used to estimate the genome-wide probability of a mutation in each of 192 possible trinucleotide contexts using SNV counts from the PCAWG dataset. The statistical procedure is described in Supplementary Methods.

##### Modeling mutation counts over an arbitrary set of positions

We conceptualized that mutations arise in a region *R* with an unknown rate whose possible values are drawn from a distribution defined by the mean and variance predicted by the deep learning network. As mutations arise, they are distributed to individual positions based on the probability that each position in *R* is mutated based on its sequence context. Let $M_{i,aX \to Yb}$ be the number of SNVs of the form $aX \to Yb$ at position *i* in region *R* in some cancer cohort of interest. Then, under a probabilistic graphical model described in Supplementary Methods, the marginal distribution over a set of possible SNVs, *I,* in a region is^31^:

$$\mathop {\sum}\limits_I {M_{i,aX \to Yb}} \sim {{{\mathrm{NegativeBinomial}}}}\left( {\alpha _R,\frac{1}{{1 + C_{{{{\mathrm{SNV}}}}} \cdot \theta _R \cdot \mathop {\sum}\nolimits_I {p_{R,aX \to Yb}} }}} \right).$$

where $\alpha _R = \mu _R^2/\sigma _R^2$ and $\theta _R = \sigma _R^2/\mu _R$ (recall *μ*_*R*_ and $\sigma _R^2$ are the mean and variance of mutation rate in region *R* estimated by the deep learning model); $p_{R,aX \to Yb}$ is the genome-wide probability of a mutation of the form $aX \to Yb$, normalized such that the probability of all possible mutations in *R* sums to 1; and *C*_SNV_ is a constant scaling factor that accounts for the difference in sample size between the cohort of interest and the training cohort.

All parameters in the distribution except *C*_SNV_ are already estimated from the training cohort. By default, *C*_SNV_ is calculated as the ratio of the number of observed synonymous SNVs in the target dataset to the number of expected synonymous SNVs in the training cohort across all genes excluding *TP53* (in which some synonymous mutations are under positive selection^4^). Thus, once the model has been trained once on the training cohort, calculating the distribution over any set of mutations in a target cohort of interest is essentially reduced to the constant time look-up of parameters. More details on the graphical model, including its extension to indels, multi-allelic variants and sets of variants that span multiple regions, are described in Supplementary Methods.

### Comparison to existing driver detection methods

We compared Dig’s performance to that of six existing methods (NBR^34^, dNdScv^4^, MutSigCV^21^, Larva^18^, DriverPower^19^ and ActiveDriverWGS^20^) over two benchmarks: accuracy of the background mutation rate models and accuracy of driver detection. The six comparison methods were chosen because they are state-of-the-art methods that (1) identify putative driver candidates by searching for mutational excess and (2) are designed to model diverse regions of the genome: tiled regions (NBR), coding sequence (dNdScv and MutSigCV) and non-coding elements such as enhancers (Larva, ActiveDriverWGS and DriverPower). All methods were run with default parameters.

#### Comparing background mutation rate models

We compared the variance explained of observed SNV counts between models. Variance explained is the proportion to which a mathematical model accounts for variation in a dataset, which we calculated as the square of the Pearson correlation coefficient between predicted and observed SNV counts, as in previous work^16^. To ensure sufficient benchmarking power, we restricted comparisons to 16 cancer types in the PCAWG dataset with >1 million mutations because the variance-explained statistic becomes deflated when observed counts are low in a discrete system (Supplementary Methods). Comparisons were performed over non-overlapping 10-kb regions of the genome (Dig versus NBR), non-synonymous SNVs in coding sequences (Dig versus dNdScv versus MutsigCV) and the non-coding elements enhancers and long and short non-coding RNAs (Dig versus Larva versus DriverPower) (ActiveDriverWGS was not included because it does not output its internal estimates of mutation counts). We chose enhancers and non-coding RNAs because they are non-coding elements that all three methods could analyze and are sufficiently far from coding sequence that synonymous mutations cannot be used in general to estimate the neutral mutation rate. To control for confounding from element length (longer elements have more mutations on average than shorter elements), we restricted the analysis to genes 1–1.5 kb in length (*n* = 3,740) and non-coding elements 0.5–1 kb in length (*n* = 7,412). Additional details of region selection are described in Supplementary Methods.

#### Comparing driver element identification accuracy

##### Coding models

We compared the sensitivity, specificity and F1-score (harmonic mean of sensitivity and specificity) for driver gene detection from coding sequence mutations among Dig, MutSigCV and dNdScv across the 32 PCAWG cohorts (melanomas and hematopoietic cancers were excluded as in previous comparisons^19^). We additionally compared power over the 16 whole-exome sequenced cohorts from Dietlien et al.^10^ (excluding hematopoietic cancers as above). Details of both comparisons are provided in Supplementary Methods.

##### Non-coding models

We compared the sensitivity, specificity and F1-score for driver non-coding element identification from non-coding SNVs among Dig, DriverPower, Larva and ActiveDriverWGS^20^ across the 32 PCAWG cohorts (excluding melanoma and hematopoietic cancers as above). We chose to compare to these three methods because they are recently introduced methods for non-coding driver element identification that rely on neutral mutation models to test for selection. Details are provided in Supplementary Methods.

### Power analysis

We conservatively simulated the power of Dig to detect driver SNVs at different carrier frequencies across enhancers and non-coding cryptic splice sites under the pan-cancer mutation map using a Monte Carlo approach described in Supplementary Methods.

### Quantifying selection on cryptic splice SNVs

#### Curation of predicted splice SNVs

From SpliceAI^40^, we obtained a list of every possible SNV in the body of 17,816 autosomal genes with predicted impact on splicing (that is, SpliceAI Δ score) >0.2. Predicted splice-altering SNVs were separated into canonical (altering positions 1 bp or 2 bp 5′ or 3′ to an exon boundary) from cryptic splice SNVs (all other SNVs excluding sites that were 5 bp 3′ to an exon boundary that had been included in the definition of ‘essential splice sites’ considered by Martincorena et al.^4^— excluded to ensure that any enrichment we observed was independent of enrichment reported in that work). SNV positions were assigned based on the GENCODE V24 list of basic transcripts. Cryptic splice SNVs were further divided into coding SNVs (defined as synonymous SNVs common to each transcript of a gene) and intronic SNVs (defined as SNVs not falling within any coding sequence of any transcript).

#### Enrichment of coding mutations and splice SNVs in PCAWG

Dig was applied with default settings to the following sets of mutation from the PCAWG cohort in each of 17,815 genes for which we had predicted splice SNVs: synonymous SNVs, missense SNVs, nonsense (stop-gained) SNVs, coding indels, canonical splice SNVs and cryptic splice SNVs. Mutation enrichment was defined as the ratio of the observed mutations to expected mutations (this statistic is conceptually similar to the selection coefficient reported for coding mutations by dNdScv). *P* values for a gene set and mutation type were exactly calculated by convolving the mutation-type-specific negative binomial distributions for each gene in the gene set and summing the upper-tail probability that at least the number of observed mutations occurred by chance. We used a Monte Carlo simulation approach to estimate the 95% CIs of enrichment within a set of genes and given mutation type (Supplementary Methods). To further assess mutational enrichment, we directly compared the rate of mutations in TSGs and oncogenes to the rate in genes not in the CGC (Supplementary Methods). The excess of SNVs in TSGs in the CGC stratified by function (missense, nonsense, canonical splice and non-coding canonical splice) was calculated as the difference between the number of mutations observed and the number expected. The relative contribution for each functional category was defined as the excess for that category normalized by the sum of the excess across all categories. The 95% CI for the contribution of each category was calculated using a Monte Carlo approach (Supplementary Methods).

#### Genes enriched for non-canonical cryptic splice SNVs

In each of the 37 PCAWG cohorts, we identified genes with a significant burden of non-canonical cryptic splice SNVs as quantified by Dig. We considered two sets of genes: (1) all TSGs in the CGC (*n* = 283) and (2) all autosomal genes with predicted splice SNVs (*n* = 17,815). The significance threshold was defined per cancer as FDR *q* < 0.1 corrected for the number of tests (*n* = 283 or *n* = 17,815). We excluded genes where multiple SNVs contributing to the burden were observed in a single sample. We used a bootstrap method to determine whether predicted cryptic splice SNVs observed in TSGs with a significant burden were enriched for high predicted impact on splicing (Supplementary Methods).

#### Analysis of alternative splicing events in RNA-seq data

We obtained RNA-seq data for eight samples carrying deep intronic predicted cryptic splice SNVs (that is, distance to nearest exon boundary >20 bp) in TSGs with a significant burden of predicted non-coding cryptic splice SNVs and 41 control samples without a cryptic splice SNV. For each carrier–control pair of the same cancer type, we performed differential splicing analysis using LeafCutter as described by Li et al.^41^. Further details of the analysis are provided in Supplementary Methods.

### Quantifying mutational excess in promoters and 5′ UTRs

#### Discovery of elements with a burden of mutations

Dig with default parameters was used to evaluate the PCAWG cohort (excluding hypermutated samples with >3,000 coding mutations) for mutational excess within two sets of regions: (1) indel excess within promoters previously defined by the PCAWG consortium^5^ (*n* = 19,251) and (2) SNV and indel excess within 5′ UTRs of TSGs (*n* = 106) and oncogenes (*n* = 95) in the CGC that spanned multiple exons of the canonical transcripts of genes (as defined by the UCSC genome browser for GRCh37); we additionally included the splice regions of the 5′ UTRs in our analysis, defined as the 20 bp bordering the start or end of an exon. The significance threshold was defined per cancer as FDR *q* < 0.1 corrected for the number of tests (*n* = 19,251 or *n* = 201).

#### ELF3 5′ UTR mutations in the Hartwig Medical Foundation cohort

We downloaded somatic mutations observed in the Hartwig Medical Foundation metastasis cohort^50^ from their online data portal (https://database.hartwigmedicalfoundation.nl/), excluding skin and hematopoietic tumors. Because we could only download mutations specific to a gene, we did not quantify burden with Dig. Rather, we directly compared the rate of SNVs in the 5′ UTR, first intron and 1-kb upstream region of *ELF3* to the rate of synonymous mutations in *ELF3* using a two-sided Fisher’s exact test.

#### Analysis of expression levels

We obtained gene expression levels (FPKM) and gene-level copy number estimates from the PCAWG data portal for all tumors for which RNA sequencing was performed. For a gene of interest, we applied a fixed-effects linear regression model to residualize the expression values for gene-level copy number per sample and the interaction between gene-level copy number and the cancer project that originally generated the RNA-seq data. We then normalized the residual expression values to have mean zero and unit variance across all samples and compared the normalized values between mutation carriers and non-carriers using a two-sided Mann–Whitney *U*-test.

### Driver gene prediction in whole-exome and targeted sequenced samples

#### Mutational excess in ‘long-tail’ driver genes

For each of the ten cancer types for which we compiled SNVs from whole-exome and targeted sequenced cohorts, we assembled a list of known driver genes identified in any of three recent pan-cancer driver gene discovery efforts^7,10,11^ (we required genes be discovered with FDR < 0.1, the significance threshold common across the driver element detection literature) that were also common to all whole-exome and targeted sequenced samples (*n* = 69 oncogenes and *n* = 56 TSGs). For a given cancer, we considered ‘long-tail’ genes to be driver genes that were not on the list of known driver genes for the given cancer (that is, they were driver genes associated with other cancers). Dig was then used to quantify mutational excess in those long-tail genes. Because synonymous mutations were not available from the targeted sequenced samples, we instead used missense mutations with CADD phred score <15 to estimate the scaling factor that adapted the somatic mutation maps trained on PCAWG cohort to the meta-cohorts (details in Supplementary Methods). We directly estimated the *P* value of the mutational burden long-tail genes by convolving the neutral mutation distributions for each individual gene and calculating the upper-tail probability of at least the number of observed mutations across all genes occurring by chance under the null distribution. We calculated 95% CIs of excess mutations using the same Monte Carlo approach as in our analysis of cryptic splice SNVs. Excess rate per sample was calculated as the number of excess SNVs divided by the number of samples in the cohort for a given cancer type.

#### Identification of putative driver genes

We used Dig to identify individual genes with an excess of mutations in two cases: (1) in our meta-cohorts, testing 69 oncogenes for an excess of activating SNVs and 56 TSGs for an excess of pLoF SNVs (these were the set of known driver genes common to all whole-exome and targeted sequenced cohorts); and (2) in the exome-sequenced cohorts alone, testing 19,210 autosomal genes for an excess of pLoF SNVs. In each case, significance was defined as FDR *q* < 0.1 for the number of genes tested.

### Box plot elements

All box plots have the following elements: center line, median; box limits, upper and lower quartiles; and whiskers, 1.5× interquartile range. Where shown, points depict all points used to construct the box-plot.

### Reporting Summary

Further information on research design is available in the Nature Research Reporting Summary linked to this article.

## Online content

Any methods, additional references, Nature Research reporting summaries, source data, extended data, supplementary information, acknowledgements, peer review information; details of author contributions and competing interests; and statements of data and code availability are available at 10.1038/s41587-022-01353-8.

## Supplementary information

Supplementary Information Supplementary Methods, Supplementary Notes 1–7 and Supplementary Figs. 1–14

Reporting Summary

Supplementary Table Supplementary Tables 1–28

Supplementary Data Regions and model predictions for each fold of each cancer for which a deep learning model was trained. Regions that were filtered are also listed along with their respective model predictions.

## Data Availability

Data generated as part of this study are available as supplementary tables or from http://dig-cancer.csail.mit.edu/. Browsable mutation maps for 37 cancer types are provided at https://resgen.io/maxsh/Cancer_Mutation_Maps/views. PCAWG data are available from https://dcc.icgc.org/releases/PCAWG/. Hartwig Medical Foundation data are available from https://database.hartwigmedicalfoundation.nl/. Whole-exome sequencing data compiled by Dietlein et al. are available from http://www.cancer-genes.org/. Targeted sequencing data are available from https://www.cbioportal.org/. The list of genes in the Cancer Gene Census is available at https://cancer.sanger.ac.uk/cosmic/download.

## Code availability

The method described in this manuscript (Dig) is available as a package hosted on the conda repository (https://anaconda.org/mutation_density/digdriver). Installation instructions and documentation are available at https://github.com/maxwellsh/DIGDriver/wiki. The Dig source code is available on GitHub (https://github.com/maxwellsh/DIGDriver). All other code used in this study is available from the authors upon reasonable request.
