# Supplementary material for: Genome-wide mapping of somatic mutation rates uncovers drivers of cancer
Source: Nat Biotechnol. 2022 Jun 20;40(11):1634–43. doi: 10.1038/s41587-022-01353-8 (PMC9646522; doi:10.1038/s41587-022-01353-8)
Supplement: Supplementary file 1 — Supplementary Methods, Supplementary Notes 1–7 and Supplementary Figs. 1–14 [file 41587_2022_1353_MOESM1_ESM.pdf]

nature portfolio

https://doi.org/10.1038/s41587-022-01353-8

Supplementary information

Genome-wide mapping of somatic mutation rates uncovers drivers of cancer

In the format provided by the authors and unedited

Supplementary information for:

Genome-wide somatic mutation rate maps uncover drivers of cancer

Maxwell A. Sherman, Adam Yaari, Oliver Priebe, Felix Dietlein, Po-Ru Loh, Bonnie Berger

Contents

Supplementary methods 2
- **Technical details of Dig's deep-learning framework** 2
- **Technical details of Dig's probabilistic graphical model** 4
- **Associating epigenetic structure to mutation density with feature maps** 9
- **Additional details about the comparison of mutation rate models** 11
- **Constructing a genome-browser of genome-wide mutation rate estimates** 15
- **Additional details about quantifying selection on cryptic splice SNVs** 16

Supplementary notes 20
1. **Insights into mutation rate prediction accuracy from feature maps** 20
2. **Comparison of cancer driver detection methods** 20
3. **Variance estimation in deep neural networks with Gaussian processes** 21
4. **Additional details on alternative splicing analysis with LeafCutter** 22
5. **Investigation of mutational burden in *ELF3* 5' UTR** 23
6. **Functional correlates of mutations in rare driver genes** 25
7. **Preliminary analysis of enhancer networks** 26

Supplementary Figures 27

Supplementary methods

Technical details of Dig's deep-learning framework

Deep-learning network architecture

Convolutional neural network

The CNN architecture is as follows: it contains 4 convolutional blocks with 2 batch normalized convolutional layers and ReLU activation. The first block reduces the 735×100 input tensor to 256×50 with 256 channels and a double stride. The following blocks are ResNet-style residual blocks which maintain their input dimension to facilitate residual connections with 256, 512, and 1024 channels respectively. Between each of the 3 residual blocks there is a double stride (ReLU activated and batch normalized) convolutional layer, which reduces the tensor length by half and doubles its height with additional channels. The output of the last residual block is flattened (and optionally concatenated with the two-flanking region counts) and passed through 3 fully connected (FC) layers. The first two FC layers are ReLU activated and reduce the dimensionality of the vector to 128 and 16 dimensions respectively. The last FC layer performs the final regression that predicts the SNV count in the 10kb region via a linear function. The CNN architecture was implemented in PyTorch[1].

Gaussian process

The Gaussian process is a sparse, inducing-point GP[2] with a radial basis function kernel that takes as input the final 16-dimensional feature vector of the trained CNN and non-linearly predicts both the mean and variance of the neutral mutations in the associated 10kb region. The GP architecture was implemented in GPyTorch[3].

Deep-learning model training

Filtering of 10kb regions

To avoid training the model over regions with inaccurate mutation counts due to technical noise, we removed regions likely to contain spurious mutation counts, defined as windows where less than 50% of the 36mers uniquely mapped back to that region or regions in the top 99.99th percentile of mutation counts.

Model training

The CNN and GP were trained sequentially. First, the CNN was trained for 20 epochs with a batch size of 128 samples, using the Adam optimizer to minimize mean squared error loss to the observed mutation counts in each training window. For training, the input data was additionally divided via an 80-20 split into training data and validation data (thus for each fold, 64% of the genome was used for training, 16% for validation, and 20% for held-out prediction). To avoid overfitting the data, the epoch from which the trained CNN was selected was determined by highest validation R-squared accuracy to observed counts in the validation-set across all CNN epochs. Once the CNN was trained, the final 16-dimension feature vector for each training window was passed as input to the Gaussian process which was trained to predict the observed mutation counts in each training window by minimizing a multivariate normal loss function with the Adam optimizer. The GP was optimized with 400 inducing points for 50 iterations. Due to the inherent variability in gradient-based optimization, we ran the GP five independent times and calculated the ensemble average of the mean and variance predictions from each of the individual runs on the held-out set of regions. These ensemble predictions were then used as the mean and variance estimates for each 10kb region. For each fold, we also predicted mean and variance of mutation counts in windows filtered prior to training. The ensemble average across all GP runs and all folds were used as the mean and variance estimates for these regions.

Some random initializations of the GP would fail to converge (defined as a decrease in R-squared accuracy of more than 0.03 compared to the final accuracy of the trained CNN).

When this occurred, the GP was restarted up to 3 times to achieve a successful convergence. If after 3 attempts, the GP had not successfully converged, the number of inducing points was reduced by 100 and the GP given another 3 attempts to converge. This process continued until successful convergence or a reduction to zero inducing points. If a GP failed to converge in all 12 attempts, the CNN was reinitialized to generate a new set of feature vectors.

Technical details of Dig's probabilistic graphical model

We derived a probabilistic method to estimate a distribution over the number of SNVs and indels observed at a set of positions in a dataset of interest given the kilobase-scale estimated mutation rate μ_R and estimation uncertainty σ_R^2 along with the sequence context likelihood estimates. We refer to this method as Dig.

Estimating genome-wide likelihood of mutation from sequence context

Let $V_{aX\to Yb}$ be the number of times that the nucleotide context a, X, b is observed with X mutated to the nucleotide Y in the training cancer cohort and let N_{aXb} be the number of times the trinucleotide a, X, b occurs in the genome, where $a, X, b \in \{A, C, T, G\}$ and $Y \in \{A, C, T, G\} \backslash X$. Then the genome-wide likelihood of the mutation $aX \to Yb$ was estimated as $\Pr(aX \to Yb) = p_{aX\to Yb} = \frac{V_{aX\to Yb}}{n \cdot N_{aXb}}$ where n is the total number of samples in the training cohort. While we chose to use a trinucleotide model in this work, any model that predicts the likelihood of a mutation solely from sequence context could be used, for example a pentanucleotide model or composite likelihood model[4].

Passenger model for SNVs

Let $M_{i,aX\to Yb}$ be the (discrete) number of mutations of the form $aX \to Yb$ at position i; X_R be the number of mutations in a kilobase-scale region R; and λ_R be the *rate* at which mutations occur

in the region R. We assume the following generative model for how mutations accumulate within the genome for a cancer of interest (**Supplementary Fig. 1**):

$$\Pr\left(M_{i,aX\to Yb}=k,X_R,\lambda_R\right)=\Pr\left(M_{i,aX\to Yb}=k\mid X_R\right)\cdot\Pr(X_R|\lambda_R)\cdot\Pr(\lambda_R)$$

where

$$\lambda_R\sim\text{Gamma}(\alpha_R,\theta_R)$$

$$X_R|\lambda_R\sim\text{Poisson}(\lambda_R)$$

$$M_{i,aX\to Yb}|X_R\sim\text{Binomial}\left(X_R,p_{R,aX\to Yb}\right).$$

The parameters α_R and θ_R are the shape and scale parameters of a gamma distribution, respectively. The parameter $p_{R,aX\to Yb}$ is the normalization of $p_{aX\to Yb}$ such that the probability of all possible mutations in R sums to one.

While the value of $M_{i,aX\to Yb}$ is observed in a cancer cohort of interest, X_R and λ_R are unknown parameters, and thus must be integrated out for the model to be of practical use. We showed previously that the generative model is an extension of the classic Poisson-Gamma distribution *and* that the marginal distribution $\Pr\left(M_{i,aX\to Yb}=k\right)$ has a simple closed form[5]:

$$M_{i,aX\to Yb}\sim\text{NegativeBinomial}\left(\alpha_R,\frac{1}{1+\theta_R\cdot p_{R,aX\to Yb}}\right).$$

Moreover, assuming that SNVs arise approximately independently, this extends to any set of SNVs $I\subseteq R$ as

$$\sum_I M_{i,aX\to Yb}\sim\text{NegativeBinomial}\left(\alpha_R,\frac{1}{1+\theta_R\cdot\sum_I p_{R,aX\to Yb}}\right).$$

We employ a variational approach to estimate α_R and θ_R. In particular, values for these two parameters uniquely determine the mean and variance of a gamma distribution. Thus, mean and variance estimates for a region R uniquely determine the values for α_R and θ_R. Given μ_R and σ_R^2 estimated by our deep-learning method, the variational estimates for α_R and θ_R are:

$$\alpha_R=\frac{\mu_R^2}{\sigma_R^2}$$

$$\theta_R = \frac{\sigma_R^2}{\mu_R}.$$

Finally, since μ_R and σ_R^2 are estimated for a particular cancer cohort used for training, they must be updated to account for any differences between the training cohort and the target cohort of interest. Assuming that the mutational processes (and technical analysis of the training cohort and target cohort) are similar, the model for the training cohort can be readily adjusted to the target cohort by the introduction of a single scaling factor C_{SNV} that, intuitively, accounts for the difference in sample size between the training and target cohorts. Under this formulation, the distribution over a set of possible SNVs in a region is:

$$\sum_I M_{i,aX\to Yb} \sim \mathrm{NegativeBinomial}\left(\alpha_R, \frac{1}{1 + C_{\mathrm{SNV}} \cdot \theta_R \cdot \sum_I p_{R,aX\to Yb}}\right).$$

By default, we estimate the scaling factor as the ratio of the number of observed synonymous SNVs in the target dataset to the number of expected synonymous SNVs in the training cohort across all genes excluding *TP53* (in which some synonymous mutations are under positive selection[6]). However, C_{SNV} can be equivalently estimated by other approaches when synonymous mutations are not available or are observed in only some genes (see below and **Supplementary Fig. 12**).

The assumption of biological and technical similarity between the training and target cohort introduces an important limitation to our approach. The training and target cohorts must be carefully matched, similar to how genetic ancestry must be matched between an imputation panel and a target SNP dataset in population genetics in order for imputation of genetic variation to be accurate. The standardization of sequencing and variant calling pipelines in recent years likely minimizes the extent of technical differences between datasets. However, matching the type of cancer between the training and target cohort is crucial.

Passenger model for indels and multi-nucleotide variants

The indel model is identical to that of the SNV model with two exceptions. First, we assume a uniform distribution of indels independent of sequence context, as has been assumed in previous works[6]. Thus in the negative binomial distribution above, $\sum_I p_{R,aX\to Yb}$ is replaced by the uniform mutation probability $|I|/|R|$ where $|\cdot|$ denotes the total number of genomic positions in I and R. The uniform assumption of indels could readily be replaced with a probability distribution based on indel type, size and homology [7], but we do not pursue that extension here. Second, the scaling factor for indels, C_{indel}, is estimated as the ratio of the number of indels observed in the target dataset to the number of expected indels in the training dataset across the coding sequence of all genes not in the Cancer Gene Census. We treat multi-nucleotide variants (MNVs) as indels.

We tested estimating μ_R and σ_R^2 independently for SNVs and indels using separate deep learning models for the two types of mutations. We found that direct estimation of these parameters for indels resulted in a less accurate indel model than using the SNV estimates as a proxy for indel estimates. We suspect this is due to the fact that indels occur an order of magnitude less frequently than SNVs and thus there are too few observed indels in the training cohort for the deep-learning model to build an accurate prediction function. As sample sizes become larger, we expect that directly training a deep-learning model to predict indels will yield more accurate predictions.

Extension to mutations spanning multiple kilobase-scale regions

We take two approaches to extend the above passenger models to account for sets of mutations that span multiple kilobase-scale regions.

- Approach 1: approximate the distribution across the regions by extending the variational estimation of α_R and θ_R. Specifically, let $R' = \{R_1, \dots, R_n\}$ be the set of

regions in which a set of mutations occur. Then we estimate $\mu_{R'} = \sum_{i=1}^{n} \mu_R$ and $\sigma_{R'}^2 = \sum_{i=1}^{n} \sigma_R^2$, and $\alpha_{R'}$ and $\theta_{R'}$ are then estimated as above from $\mu_{R'}$ and $\sigma_{R'}^2$.

- Approach 2: exactly estimate the distribution across the mutation set by convolving the distributions arising from the subset of mutations in each $R_i \in R'$.

Approach 1 is computationally efficient and accurate so long as the mutation rate estimates across $\{R_1, \ldots, R_n\}$ are sufficiently similar. Thus approach 1 is preferred when R' is composed of a small number of contiguous (or nearly contiguous) regions and is the default implemented algorithm. When R' is composed of regions with highly variable mutation rates, approach 1 is likely to either over- or under-estimate the passenger mutation rate, leading to improperly calibrated p-values. In this case, approach 2 will provide accurate estimates but requires more computation due to the convolution operation.

Testing mutational burden across a set of candidate mutations using an existing mutation map

The steps to estimate selection using Dig are as follows:

User steps:

1. Download a mutation map for the cancer matching the cancer type of the dataset of interest.
2. Provide the mutation dataset of interest and define the set I of possible mutations. I can be defined as any set of genomic intervals (contiguous or noncontiguous) or any set of possible SNVs anywhere in the genome.

Software steps:

3. The mutation likelihoods $p_{R,aX\rightarrow Y,b}$ and p_{indel} are calculated as described above for each mutation set. The nucleotide sequence of R is extracted from the reference genome.
4. The SNV and indel scaling factors are estimated for the cohort of interest.

5. The p-value of the number of SNVs and indels observed in the cohort of interest for each mutation set are calculated using the negative binomial distributions defined above as the null models. In this work, we calculated the P-value as the upper-tail probability of the observed mutation count, applying a mid-P correction to account for the discrete data.
6. The p-values for the SNVs and indels are combined via Fisher's method.

For this study, we used the mutation maps trained using both epigenetic tracks and flanking mutation counts to test for burdens of mutations. These are also the maps we have made publicly available.

Associating epigenetic structure to mutation density with feature maps

To investigate the underlying features the deep learning model considered when predicting mutation rates, we added another layer of computation between the input epigenetic matrix and the CNN to serve as feature maps. Feature maps are a tool used in computer vision tasks to detect which regions of an image the model uses to perform prediction[8]. We used this technique to evaluate which epigenetic patterns the CNN exploited to predict mutation rates. To reduce the potential for noise, we applied this technique to input matrices encoding 50kb regions.

Feature map generation

An additional two-layered network was added between the input matrix and CNN to force the model to attend to the subset of most salient input sub-regions and compute the feature maps. In the attention augmented CNN, the input matrix was first passed through two convolutional layers preserving the input dimensionality (stride length 1, kernel sizes 5 and then 3) with ReLU activations. Subsequently, the output of the two layers was passed through a row-wise Softmax function that had the effect of making most entries in the matrix close to zero with sparse values

close to one. The resulted “feature map” matrix was then element wise multiplied with the original input and passed on to the downstream CNN. This had the effect of setting most entries in the original epigenetic matrix to near zero, thus forcing the CNN to rely only on the small subspace of the input that was not zeroed out. The optimization process compels the feature maps to attend to the features of the input matrix most relevant for the prediction process.

Extraction of epigenetic content of feature maps via dimensionality reduction and clustering

While the feature maps have the theoretical ability to attend to any regions of the input matrix, in practice we found they almost always attended to a large set of epigenomic features (rows) in a small set of contiguous columns (genomic positions), zeroing out most values outside of these columns. We extracted and summarized the epigenetic content of each of these attention columns through the following approach: 1) in each 50kb window, we extracted the largest contiguous set of columns such that each column contained at least 10 cells with a non-zero entry. This contiguous set of columns was defined as an “attention super-column”. 2) Each attention super-column was reduced to an 8-dimensional vector by averaging together tracks of the same epigenetic type per column (DNase, H3K27ac, H3K27me3, H3K36me3, H3K4me1, H3K4me3, H3K9ac, and H3K9me3) and taking the maximum value across each row. 3) The vectors were normalized and projected into a two-dimensional subspace for clustering and visualization via a Uniform Manifold Approximation and Projection (UMAP) transformation (Python UMAP-learn package; # neighbors: 30, minimum distance: 0, # components: 2). 4) Spectral clustering (Python Sklearn package) was applied to the two-dimensional subspace to identify attention super-columns with similar epigenetic content. The clustering consistently identified five distinct clusters across cancer cohorts (**Extended Data Figure 2**).

Connecting feature map epigenetic clusters to functional annotations

To determine whether the attention super-columns in a cluster represented a functional epigenetic structure, we extracted the average Epilogos[9] signature vector per attention super-column and examined whether the Epilogos signatures were consistent within a cluster. Epilogos is a summary of the functional epigenetic states across 111 tissue types as inferred by the ChromHMM method.

Connecting feature map epigenetic clusters to mutation rates

We extracted the mutation count for the 50kb window in which each attention column occurred. We computed the mean and standard deviation of mutation counts across the attention column clusters.

Additional details about the comparison of mutation rate models

Deflation of variance explained statistic in low count scenarios

In discrete stochastic systems, random stochasticity of events when event rate is low results in deflation of the variance explained statistic. The characteristic arises because a discrete system generally has a fractional expected value but observations must take on integer values. Thus, even if a model perfectly predicts the expected value, it will explain relatively little variance if the difference between the fractional expected value and possible observed values is of similar magnitude to the possible observations (e.g., expected value of 0.5 versus possible observed values of 0 or 1). Intuitively, for a discrete process with event rate <1, the expected value will be a real value between zero and one but the observed count will be an integer (0, 1, 2, etc.); thus, the true expected value will explain relatively little variance of observed data because the observed values almost always deviate substantially from the expected value.

Tiled regions

We compared the variance explained (square of the Pearson correlation coefficient) in SNV counts within 10kb windows tiled across the genome between Dig and NBR [10]. NBR is, to our knowledge, the only method that has been previously used to build passenger mutation rate models in kilobase-scale regions tiled across the genome. However, code for running the NBR method is not currently publicly available. For each cancer, the NBR model was trained on the same regions used to train our deep-learning model (excluding regions with 36mer mappability <50% and regions in the top 99.99th percentile of mutation count). The regions excluded from training were also excluded when calculating the variance explained statistic. We also assessed the variance explained of SNV counts in 1Mb regions by our method and NBR (restricted to 1Mb regions with >50% 36mer mappability). To estimate the expected mutation count in each 1Mb region, we summed together the estimates of each non-overlapping 10kb window within the 1Mb region.

Coding sequence

We compared the variance explained in *nonsynonymous* SNV counts between Dig and two widely used methods that generate nonsynomous SNV passenger mutation models: MutSigCV[11] and dNdScv[6]. Both MutSigCV and dNdScv utilize the synonymous mutations observed in each gene to estimated gene-specific passenger mutation rates. Variance explained was evaluated over the coding sequence of 3,740 genes that were 1) common to all three methods; 2) between 1kb and 1.5kb in length; and 3) not in the CGC. The length restriction was imposed to prevent coding sequence length from artificially inflating variance explained since the number of mutations in a gene strongly correlates with its length.

Noncoding regulatory elements

We compared the variance explained in SNV counts between Dig and two other methods that estimate passenger mutation rates in noncoding regulatory elements: DriverPower[12] and

Larva[13]. DriverPower is optimized to estimate mutation rate within a set of regulatory elements predefined by the authors of the software; this set of elements is not easily changed. We thus evaluated variance explained in a set of 7,412 noncoding regulatory elements (enhancers, lncRNAs, and sncRNAs) between 0.5kb and 1kb in length that could be modeled by DriverPower. The length restriction was again implemented to prevent inflation of variance explained due to variance in element length. While Larva can predict mutation rate within genomic intervals, it cannot natively provide a prediction for elements that are composed of multiple, non-contiguous intervals. To circumvent this, we divided each element evaluated by DriverPower into its constituent intervals, produced a prediction for each interval separately with Larva, and summed the predictions across regions composing a single element.

Details about the comparison of driver element detection methods

Comparison of driver gene detection methods

We compared the sensitivity, specificity, and F1-score (harmonic mean of sensitivity and specificity) for driver gene detection from coding sequence mutations between Dig, MutSigCV, and dNdScv across the 32 PCAWG cancer cohorts (melanomas and hematopoietic cancers were excluded as in previous comparisons[19]). We chose to compare to these two methods because they are widely used driver gene detection methods that rely on neutral mutation models to test for selection. An FDR significance threshold of 0.1 was applied for all methods and cohorts. A true-positive driver gene was defined as any gene in the Cancer Gene Census (CGC)[39] that was detected as FDR significant by any of the methods in a given cohort. A false-positive was defined as any gene identified as FDR significant that was not in the CGC. Each method was applied to the same set of 16,794 genes. Both SNVs and indels were used to identify potential driver genes. We additionally compared power over the 16 whole-exome sequenced cohorts from Dietlien et al. (excluding hematopoietic cancers as above). The larger

cohort sizes enabled the approximation of receiver-operator characteristic curves for the methods. The curves were approximated because genes in the CGC were used as a proxy for true-positives (that is, a gene not in the CGC may still be a true-positive driver but would be counted as a false-positive in this analysis). Because of the approximated nature of these curves, we visualized the results as false-positive counts vs true positive counts rather than the standard false-positive vs true-positive rates, following precedent from Dietlein et al. The power of a method was quantified as the area under these approximated receiver-operator characteristic curves.

Comparison of noncoding driver element detection methods

We compared the sensitivity, specificity, and F1-score for driver noncoding element identification from noncoding SNVs between Dig, DriverPower, Larva, and ActiveDriverWGS[20] across the 32 PCAWG cancer cohorts (excluding melanoma and hematopoietic cancers as above). We chose to compare to these three methods because they are recently introduced methods for noncoding driver element identification that rely on neutral mutation models to test for selection. An FDR significance threshold of 0.1 was applied for all methods and cohorts. A true-positive driver element was defined as any element previously identified by PCAWG as carrying a burden of mutations[5] that was detected as FDR significant by any of the methods in a given cohort. A false-positive was considered any FDR significant element that was not previously identified by PCAWG as having a burden of mutations. This comparison was conservative (biased against our approach) for two reasons: 1) The other three methods were previously applied to the PCAWG dataset to generate the set of putative driver elements that we then used as a gold standard for the same samples; and 2) we restricted the analysis to SNVs because not all methods we compared to could accept indels. Indeed, our approach is the only approach that models SNVs and indels independently; the other approaches either do not model indels or model indels and SNVs as a single category.

Constructing a genome-browser of genome-wide mutation rate estimates

We used Dig to estimate mutation rates in every non-overlapping regions of size 100bp, 250bp, 500bp, 1kb, 2.5kb, 25kb, 50kb, 100kb, 250kb, 500kb and 1Mb tiled across the genome (excluding assembly gaps in the GRCh37 reference genome) for 37 PCAWG cancer types. These predictions were used to construct data structures that can be interactively visualized by HiGlass[14].

Details about power analysis

We conservatively simulated Dig's power to detect driver SNVs at different carrier frequencies across enhancers and noncoding cryptic splice sites under the pan-cancer mutation map using the following Monte Carlo approach.

For a given sample size and carrier frequency of driver mutations:

1. For each element, randomly draw a mutation rate parameter from the gamma distribution defined by mean and variance estimated by the kilobase-scale model.
2. For each element, estimate the scaling factor as the target sample size divided by the pan-cancer sample size (n=2,279) and randomly draw an observed number of mutations from a Poisson distribution with rate parameter equal to the sampled rate multiplied by the scaling factor and by the probability of an SNV in the element.
3. For each element, randomly sample the number of driver mutations from a Poisson distribution with rate parameter equal to the target sample size multiplied by the carrier frequency.
4. Count the number of elements for which the sum of the background mutations and driver mutations exceeded the Bonferroni-corrected α<0.05 threshold under Dig's

negative binomial null mutation distribution for each element. Divide the count by the total number of tested elements to estimate a detection likelihood.

5. Repeat steps 1-4 one thousand times and average the detection likelihoods across all simulations.

Additional details about quantifying selection on cryptic splice SNVs

Monte Carlo method for estimating confidence intervals of mutational enrichment.

Mutation enrichment was defined as the ratio of the observed mutations to expected mutations. We used the following Monte Carlo simulation approach to estimate the 95% confidence intervals of enrichment for a given set of genes and given mutation type.

1. For each gene, estimate the enrichment coefficient as the number of observed mutations divided by the number of expected mutations. A small pseudo-count of 1×10^{-16} was added to the numerator and denominator to prevent the enrichment from being identically zero when no mutations were observed in a gene. (This would lead to a degenerate Poisson distribution in step 3).
2. For each gene, randomly draw a Poisson rate parameter from the gamma distribution defined by the mean and standard deviation estimates of the kilobase-scale mutation rate map.
3. For each gene, randomly draw a number of “observed” mutations from a Poisson distribution with rate parameter equal to the simulated rate parameter multiplied by the enrichment coefficient and the likelihood of the mutation type occurring within the gene. Conceptually, this mutation count is simulated under the hypothesis of *positive selection* on the mutations within the gene.

4. Estimate a simulated enrichment by summing the number of simulated mutations across all genes in the set and dividing by the expected number of mutations under the null model of no enrichment.
5. Repeat steps 1-4 one thousand times and define the boundaries of the 95% confidence interval as the lower 2.5th percentile and upper 97.5th percentile of the simulated enrichments.

Additional quantification of mutation enrichment in TSGs and oncogenes

To gain additional confidence in the accuracy of our mutation enrichment estimates, we directly compared the mutation rate in genes not in the CGC to TSGs and oncogenes in the CGC using a two-sided Chi-squared test for a two-by-two contingency table. This approach recapitulated the enrichment patterns we observed using Dig. However, the Chi-squared test does not account for global mutation rate differences between genes not in the CGC and genes in the CGC; thus, the precise estimates in **Supplementary Fig. 9** are unlikely to be accurate.

Identification of individual TSGs enriched for noncanonical cryptic splice SNVs

In each of the 37 PCAWG cohorts, we identified TSGs in the CGC with a significant burden of noncanonical cryptic splice SNVs under the null model estimated by our method. The significance threshold was defined per cancer as FDR q-value<0.1 corrected for the number of tested TSGs (n=283). We excluded one significant gene, *PRDM1*, from further analysis because the observed excess mutations were attributable to a single sample.

Quantification of the pan-cancer contribution of cryptic splice SNVs to TSG driver SNVs

We calculated the excess of SNVs in TSGs in the CGC stratified by function (missense, nonsense, canonical splice, and noncoding canonical splice) as the difference between the number of mutations observed and the number expected. The relative contribution for each

category was defined as the excess for that category normalized by the sum of the excess across all categories. The 95% confidence interval for the contribution of each category was calculated using the Monte Carlo approach described above for enrichment with the following modifications:

6. In step 3: for each gene, the number of neutral mutations was also simulated from a Poisson distribution with rate parameter equal to the gamma-simulated rate parameter multiplied by the probability of a mutation occurring in the gene. Conceptually, this mutation count is simulated under the hypothesis of *neutral selection* on the mutations within the gene.

- In step 4: the excess for each gene is calculated as the difference between the number of mutations simulated under positive selection and the number simulated under neutral selection. The total excess for each mutation category is summed across all genes and the relative contribution calculated as above.

Enrichment of predicted splicing impact in noncoding cryptic splice SNVs observed in significantly burdened TSGs

We used a bootstrap method to calculate a p-value for the null hypothesis that noncanonical cryptic splice SNVs observed in the genes with a significant burden of cryptic splice SNVs had a predicted impact on splicing similar to the predicted impact of cryptic splice SNVs observed in genes not in the CGC. We calculated the median of the Δ scores randomly resampled from the observed cryptic splice SNVs in the TSGs and observed cryptic splice SNVs in genes not in the CGC ten thousand times (the number of SNVs sampled from the non-CGC set was equal to the number observed in the TSG set). We estimated the p-value as the number of times the resampled median of the non-CGC cryptic splice SNVs exceeded the resampled median of the cryptic splice SNVs observed in the TSGs.

Analysis of alternative splicing events in RNA-seq data

We obtained RNA-seq data for 8 samples carrying deep intronic predicted cryptic splice SNVs (i.e., distance to nearest exon boundary >20 base-pairs) in TSGs with a significant burden of predicted noncoding cryptic splice SNVs. This represented all such carriers with available RNA-seq data. We downloaded the STAR aligned BAM files for each donor and six randomly selection non-carriers from the same cancer cohort, and we used bedtools bamtofastq to convert these reads into FASTQ files for de novo alignment. We then ran olego[15] with the default junction database and max edit distance of 4 (flag -M 4) on each FASTQ file. Olego is specifically designed for increased sensitivity to de novo splicing in RNA-seq reads. The de novo aligned sam files were then converted to bam files, sorted, indexed, and processed for junctions by Regtools[16] for downstream analysis (input parameters: -a 8 -m 50 -M 50000). For each of the carrier-control pairs, we performed differential splicing analysis using LeafCutter as described by Li et al.[17]. The introns in each pair were clustered using the leafcutter_cluster_regtools.py script, requiring a single split read to support a junction and assuming a maximum intron length of 500Kb (input flags -m 1 -o -l 500000). Differential splicing was then evaluated using the leafcutter_ds.R script using the Gencode v19 exons provided with the software. When a gene had more than one transcript available, we used the canonical transcript as annotated in UCSC genome browser. We considered a predicted splice SNV to have strong supporting evidence if LeafCutter reported a splice cluster containing the predicted splice SNV that had significantly different usage between carrier and control ($p<0.05$) in the majority of the carrier-control pairs. If LeafCutter did not report a cluster containing the predicted splice SNV, we additionally examined the raw junction files from Regtools. We considered a predicted SNV to have some supporting evidence if junctions supporting the prediction were observed in the raw junction files. Two of the eight samples were discarded due to insufficient coverage of the gene of interest (**Supplementary Table 14**).

Supplementary notes

1. Insights into mutation rate prediction accuracy from feature maps

To gain insight into which specific epigenetic features the deep-learning model utilized to achieve its high prediction accuracy over mutation counts, we leveraged an approach that highlights input features important to the model's performance (feature maps, **Supplementary Methods**). Averaging chromatin marks of the same type (e.g., H3K27ac) across tissues revealed that the network learned to focus on localized epigenetic structures (avg. size 1526 bp; 95% CI: 1512-1540 bp) corresponding to known functional elements: transcription start sites, regions of active transcription, enhancers, repressive regulatory states, and heterochromatin to make predictions within kilobase-scale regions (**Extended Data Fig. 2**). This behavior was consistent across numerous cancers (**Extended Data Fig. 2**). The functional epigenetic structures that the network learned to recognize associated with observed somatic mutation rates in ways consistent with known epigenetic correlates of mutation rates[18] (**Extended Data Fig. 2**). For example, regions of closed chromatin exhibited high mutation rates while those of active transcription exhibited relatively low mutation rates. These results add to the growing evidence that deep-learning models can implicitly learn biological structure when trained to directly predict function from sequence[19–21].

2. Comparison of cancer driver detection methods

Because our approach identifies driver candidates by testing for selection, we compared its accuracy to other methods that also test for selection. We first compared our method's ability to identify driver genes in the PCAWG dataset against MutSigCV[11] and dNdScv[6], two widely used methods created specifically to identify genes under positive selection. Following previous works[4,12], we used the Cancer Gene Census (CGC)[22] as a conservative approximation of the

true-positive rate and found our method matched or exceeded the F1-score (a joint measure of sensitivity and specificity) of the other methods in 24 of 32 PCAWG cohorts (excluding hematological and skin malignancies[12]) (uniquely highest score in 13 cohorts; tied for highest in 11 cohorts) (**Supplementary Fig. 3**, **Supplementary Table 8**). We additionally calculated the receiver-operator curves for the top 600 genes identified by each method in the PCAWG pan-cancer cohort and found Dig systematically identified more true-positive drivers and fewer false-positives than the other methods (**Supplementary Fig. 3**), a pattern that we also observed when we additionally compared the methods across whole-exome sequenced (WES) cohorts[4] (**Supplementary Fig. 4**). We additionally found that Dig's ability to accurately recall noncoding drivers previously identified in the PCAWG dataset was comparable to that of three other burden-based non-coding driver detection methods, Larva[13], ActiveDriverWGS[23], and DriverPower[12] (**Supplementary Fig. 5**, **Supplementary Table 10**), although this analysis was biased against Dig because the other three methods were used to generate PCAWG's own set of noncoding drivers.

3. Variance estimation in deep neural networks with Gaussian processes

While it is intuitive that more accurate prediction of the expected neutral mutation rate can improve power to identify drivers, the accuracy of variance prediction also plays a crucial role, particularly in ensuring well-calibrated p-values. We previously investigated the accuracy of the CNN+GP architecture to estimate kilobase-scale mutation rates compared to other architectures in a simulated dataset (full details in Yaari et al.[5]). Here we review the results about variance because they provide additional insight into reasons underlying our methods power to detect driver events.

In brief, we generated a synthetic kilobase-scale mutation rate dataset for the PCAWG melanoma, esophageal, and stomach cancer datasets using a k-nearest-neighbors strategy. For each 50kb window, we found its 500 nearest neighbors based on mean epigenetic context

from the Roadmap Epigenomics dataset and defined the “true” mean and variance for that window to be the mean and variance of the mutation rate across the 500 nearest neighbors. We then trained methods to predict the mean and variance of each window based on a mutation count randomly simulated from a negative binomial distribution defined by that true mean and variance. We then compared the predicted mean and variance to the true mean and variance for each method (**Supplementary Fig. 14**).

The simulated dataset has an interesting feature: the variance of the mutation rate plateaus beyond a certain expected mutation rate. That is, while the expected mutation rate continues to increase, the variance of the mutation rate does not increase. An important feature of a Gaussian process is that it *nonlinearly* predicts mean and variance; the relationship between mean and variance is not imposed a priori. This has the effect of enabling the CNN+GP estimation method to learn that variance plateaus as expected mutation rate increases (**Supplementary Fig. 14c**). Thus, statistical tests remain well powered to identify outlier events even at high mutation rates. This is not the case for the linear regression methods often employed to predict the mean and variance of mutation rate. For example, negative binomial regression imposes a quadratic relationship between mean and variance: $\sigma^2 = \mu(1 + \beta \cdot \mu)$, where $\beta > 0$ is the estimated overdispersion parameter. This has the effect of forcing the variance to increase *faster than* the expected mutation rate, leading to variance estimates considerably larger than the true variance when expected mutation rate is high (**Supplementary Fig. 14c**). Thus, negative binomial regression loses power to detect outliers in high mutation rate contexts.

4. Additional details on alternative splicing analysis with LeafCutter

Of the eight predicted cryptic splice SNV carriers for which we obtained RNA-seq data (**Methods**), two carriers were discarded due to insufficient coverage either at the gene of

interest (DO222305, median coverage of *CIITA* of 17 reads) or globally (DO9074, median depth of coverage of 33). Of the remaining 6 carriers, 4 had clear evidence of alternative splicing: LeafCutter[17] reported a splicing cluster containing the predicted splice SNV with significantly different usage ($P < 0.05$) between the carrier and at least a majority (4 of 6) of the control pairs (**Supplementary Table 14**). We further investigated the remaining 2 predicted cryptic splice SNV carriers and observed that one had some evidence of alternative splicing in the raw junction file. This carrier (DO52675) had evidence of differential splicing that was not reported by LeafCutter. Specifically, by manually annotating the junction files produced by Regtools[16] with the introns defined in ENSEMBL, we observed that the carrier used an alternative site consistent with the predicted splice SNV in approximately 10% of transcripts, while the controls utilized this site in approximately 1% of transcripts. The remaining carrier (DO33392) sample did not have evidence of alternative splicing upon manual review. This may be due to the mis-spliced transcripts undergoing nonsense mediated decay; however, we did not have statistical power to evaluate this hypothesis.

5. Investigation of mutational burden in *ELF3* 5' UTR

The PCAWG consortium previously carefully reviewed noncoding mutational hotspots in the PCAWG dataset[10] and cataloged several reasons for excess mutations that were unrelated to positive selection: activation-induced cytidine deaminase (AID) activity in lymphomas, impaired nucleotide excision repair (NER) at transcription factor binding sites in melanomas, activity of endogenous apolipoprotein B mRNA- editing enzyme catalytic subunit (APOBEC) family deaminases, particularly in the in the loop region of predicted hairpin structures, and systematic short-read mapping inaccuracies leading to artefactual mutation calls. We examined whether any of these processes could be responsible for the observed enrichment of SNVs in the 5' UTR of *ELF3*.

In our analysis of the 5' UTR of *ELF3*, we specifically excluded hematopoietic tumors and melanomas, so neither AID nor NER likely account for the observed elevated mutation rate. To investigate the possible role of APOBEC at the 5' UTR of *ELF3*, we obtained the results of the ABOPEC analysis performed by the PCAWG consortium in which each observed mutation was annotated for whether it could be attributed to APOBEC. Of the six SNVs observed in the *ELF3* 5' UTR, only one was annotated as occurring in a context targeted by APOBEC; however, the sample in which that mutation occurred was not significantly enriched for APOBEC mutations of that kind nor did the mutation occur within a cluster as would be expected if it were due to APOBEC mutagenesis. We thus do not believe APOBEC likely explains the mutational excess in the *EFL3* 5' UTR. We next examined the gnomAD database[24] which both cataloged population polymorphic germline genetic variation and noted regions of the genome where mapping artefacts were present. The 5' UTR of *ELF3* was not annotated as a region with mapping artefacts by gnomAD. Moreover, of the 16 somatic mutations observed in the PCAWG and Hartwig datasets, only one affected a position also affected by a germline SNP (the canonical splice site chr1:201979836, although the mutation itself is different). The germline SNP was rare (2 alleles observed in >30000 haplotypes). Moreover, the six mutations in the PCAWG dataset were observed in five different cancer types and the ten mutations in the Hartwig dataset were observed in seven different cancer types. Thus, the enrichment cannot be attributed to a mutational process specific to one cancer type. Finally, the mutation enrichment was specific to the canonical 5' UTR of *ELF3*; enrichment was not observed in surrounding regions as was noted by PCAWG for several lncRNAs. In summary, we were unable to explain the mutation burden observed in the 5' UTR of *ELF3* by processes that had been previously noted to increase mutation rate independent of positive selection.

6. Functional correlates of mutations in rare driver genes

We investigated the functional consequences of rare mutations in three genes with known phenotypes when they act as common drivers: *MSH2* (CNS tumors), *MLH1* (CNS tumors), and *SF3B1* (liver tumors). *MSH2* and *MLH1* encode DNA mismatch repair proteins[25]; inactivation of these genes increases the spontaneous mutation rate in cells[26]. Thus, carriers of pLoF mutations in these genes are expected to have elevated mutation rates compared to non-carriers. Consistent with this expectation, CNS tumors with rare pLoF mutations in both *MSH2* and *MLH1* exhibited significantly increased mutation rates relative to non-carriers across 213 targeted sequenced genes (*MSH2*: mean 30.1 mutations in carriers vs. 3.0 in non-carriers, $P=3.8\times10^{-7}$ one-sided Mann-Whitney U-test; *MLH1*: mean 35.3 mutations in carriers vs. 3.1 in non-carriers, $P=8.8\times10^{-6}$ one-sided Mann-Whitney U-test). Further supporting the potential driver role of *MSH2* in CNS tumors, the gene also exhibited a significant burden of missense mutations (18 observed vs. 5.3 expected, $P=2.5\times10^{-5}$), and missense *MSH2* carriers also exhibited a significantly elevated mutation rate (mean 35.4 mutations in carriers vs. 3.0 in non-carriers across 213 targeted sequenced genes; $P=3.7\times10^{-12}$, one-sided Mann-Whitney U-test). The mutation rate between pLoF and missense *MSH2* carriers was not statistically distinguishable (P=0.27). *MLH1* did not carry a significant burden of missense mutations in CNS tumors, though this may reflect a lack of statistical power.

SF3B1 encodes a protein involved in the splicing of pre-mRNA molecules. Activating mutations in this gene have previously been associated with increased rates of alternative 3' splice site usage and exon-skipping events[27]. One liver tumor with a rare activating mutation in *SF3B1* had been characterized with RNA-seq. Based on a quantitative accounting of the alternative splicing events in this sample from Kahles et al.[27], the carrier was in the 89th percentile for number of alternative 3' splice events amongst TCGA liver samples (40th of 368 samples) and in the 88th percentile for exon skipping events (43rd of 368 samples), exhibiting

more than a standard deviation increase in both types of events relative to the mean across liver samples. More samples are required to achieve the statistical power necessary to conclude that *SF3B1* activating mutations in tumors in which SF3B1 is rarely mutated alter splicing systematically.

7. Preliminary analysis of enhancer networks

An analysis of the SNV and indel burden in enhancers (obtained from Nasser et al.[28]) of 725 CGC genes using Dig with default settings revealed 36 enhancers with significant (FDR<0.1) mutational burdens. To coarsely filter regions potentially affected by unmodeled local hypermutation processes, we required that observed mutations each occur in a unique sample. This filter reduced the number of enhancers to ten (**Supplementary Table 27**). Two enhancers (for *LEPROTL1* and *SRGAP3*) contained recurrent mutations (*LEPROTL1*: 8:29952919-G>A (n=7), 8:29952921-C>A,G,T (n=5); *SRGAP3*: 3: 8486222-G>C,T (n=6)); however, it is possible that these mutational hotspots could result from *APOBEC* mutagenesis or mapping artefact[10]. Carriers of mutations in several enhancers demonstrated significant (P<0.05) or nearly-significant (P<0.1) differences in expression compared to non-carriers (not corrected for multiple hypothesis testing). For example, carriers of mutations in the *NCOR2* enhancer (12:125422682-125425761) had a nearly significant decrease in expression (P=0.078). However, expression did not always change in a direction consistent with the known or predicted function of the gene in tumorigenesis. For example, carriers of indels in the *MSI2* enhancer (17:54992281-54993673) had decreased *MSI2* expression (P=0.0081) based on carrier tumors from kidney, rectum, and ovary; however, *MSI2* is a known oncogene in hematopoietic cancers. More follow-up analysis will be necessary to determine whether the mutational enrichment constitutes positive selection or unaccounted for neutral mutational processes.

Supplementary Figures

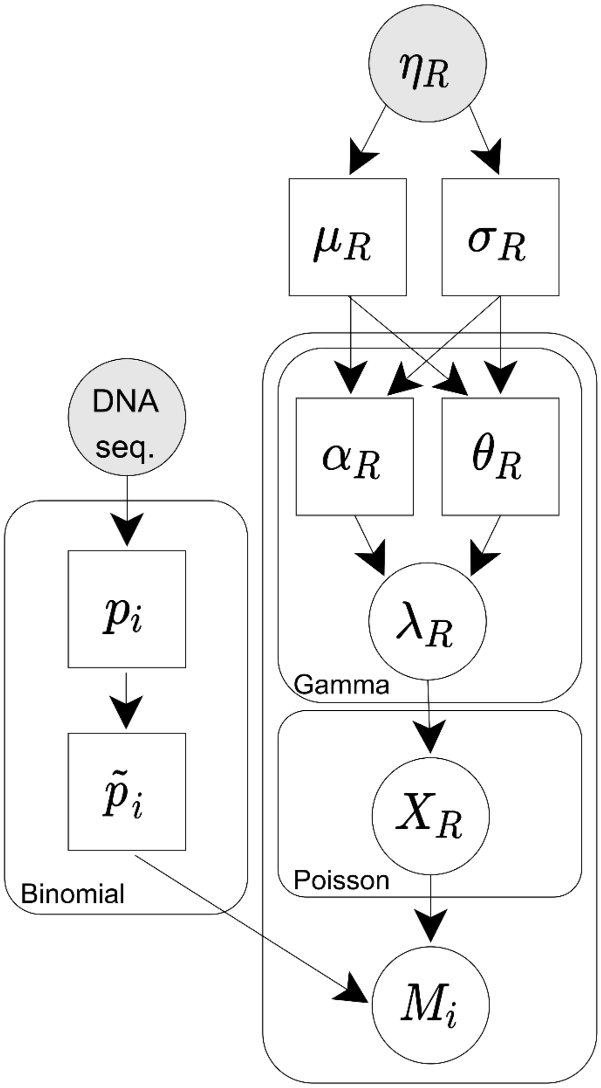

Supplementary figure 1: Plate diagram of the probabilistic model that Dig uses to model the number of neutral mutations (M_i) in an element of interest. η_R: observed data used as input to Dig's deep-learning model (chromatin modifications and, optionally, flanking mutation counts) to estimate regional neutral mutation parameters for region R. μ_R and σ_R: mean and standard deviation estimates of the neutral mutation rate in region R. α_R and θ_R gamma distribution shape and scale parameters, respectively. λ_R gamma-distributed mutation rate parameter for region R. X_R poisson-distributed mutation count in region R. DNA seq.: the DNA sequence from the human reference genome. p_i: genome-wide likelihood of a mutation in a given DNA context centered at position i. $\tilde{p}_i$: likelihood of mutation based on sequence context centered at position i normalized such that $\sum_{i \in R} \tilde{p}_i = 1$. See **Methods** for additional details.

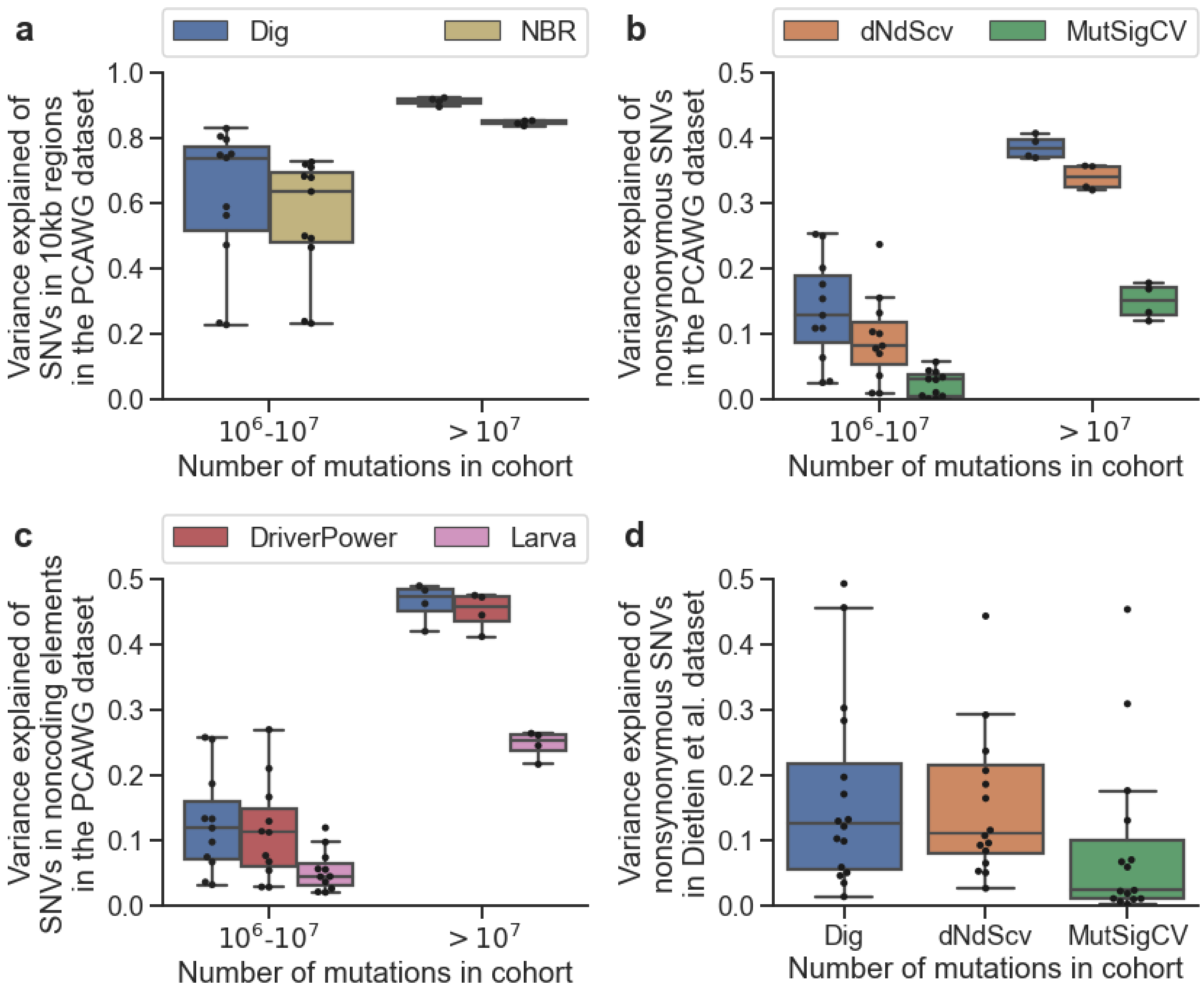

Supplementary figure 2: Comparison of variance explained of SNV counts across methods, annotations, and cohorts. a, Variance explained of SNV count in 10kb regions tiled across the genome by Dig and NBR[10] in N=16 PCAWG cancer cohorts with >1 million SNVs (excluding hemopoietic tumors, for which NBR failed to converge). Regions in which <50% of 36mers are unique are excluded as are regions in the 99.99th percentile of mutation count. **b,** Variance explained of nonsynonymous SNV count in genes 1-1.5kb in length (n=3,740 genes) in N=16 PCAWG cancer cohorts. **c,** Variance explained of SNV count in enhancers and noncoding RNAs (long and short) 0.5-1kb in length (n=7,412 noncoding elements) in 16 PCAWG cancer cohorts. **d,** as **b** for 16 whole-exome sequenced cancer cohorts from Dietlein et al.[4]. Box-plot elements defined in Methods. Number of samples and mutations in each cohort are in **Supplementary Table 4** (**a**), **Supplementary Table 5** (**b**), **Supplementary Table 6** (**c**) and **Supplementary Table 28** (**d**).

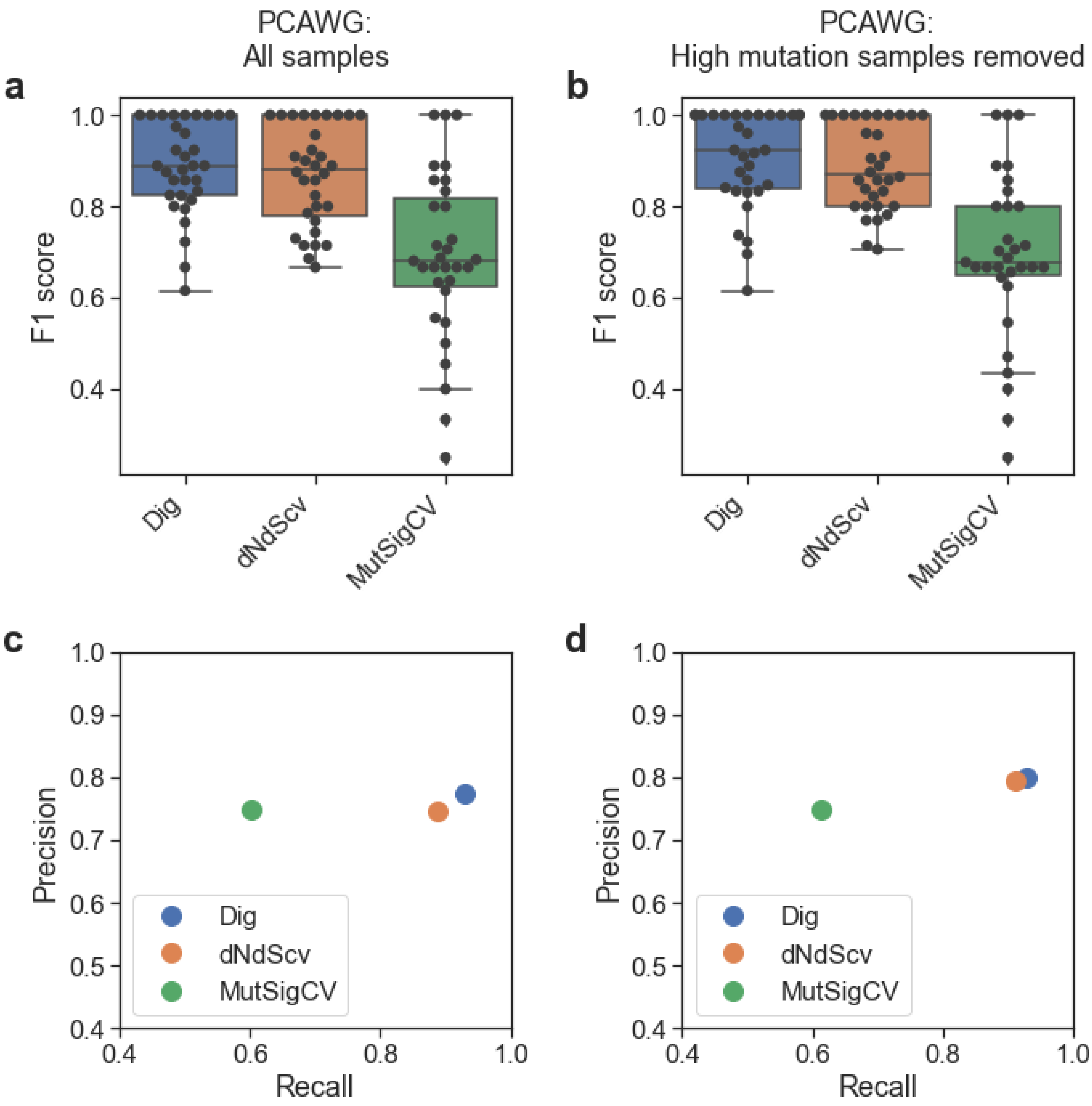

Supplementary figure 3: Precision-recall comparison of gene driver methods in the PCAWG cohort. a,b F1-score (harmonic mean of precision and recall) in N=32 PCAWG cohorts (melanoma and hematopoietic tumors were excluded as in previous work[12]) across 16,794 genes common to the three methods. Precision and recall were calculated using genes in the Cancer Gene Census as a conservative true positive set. **a,** All samples. **b,** Excluding samples with >3000 coding mutations and restricting the total number of mutations per sample per gene to 3 (default filtering options for dNdScv). **c,d** Recall and precision measured across all N=32 PCAWG cohorts for **c,** all samples and **d,** samples with <3000 coding mutations. Box-plot elements defined in Methods.

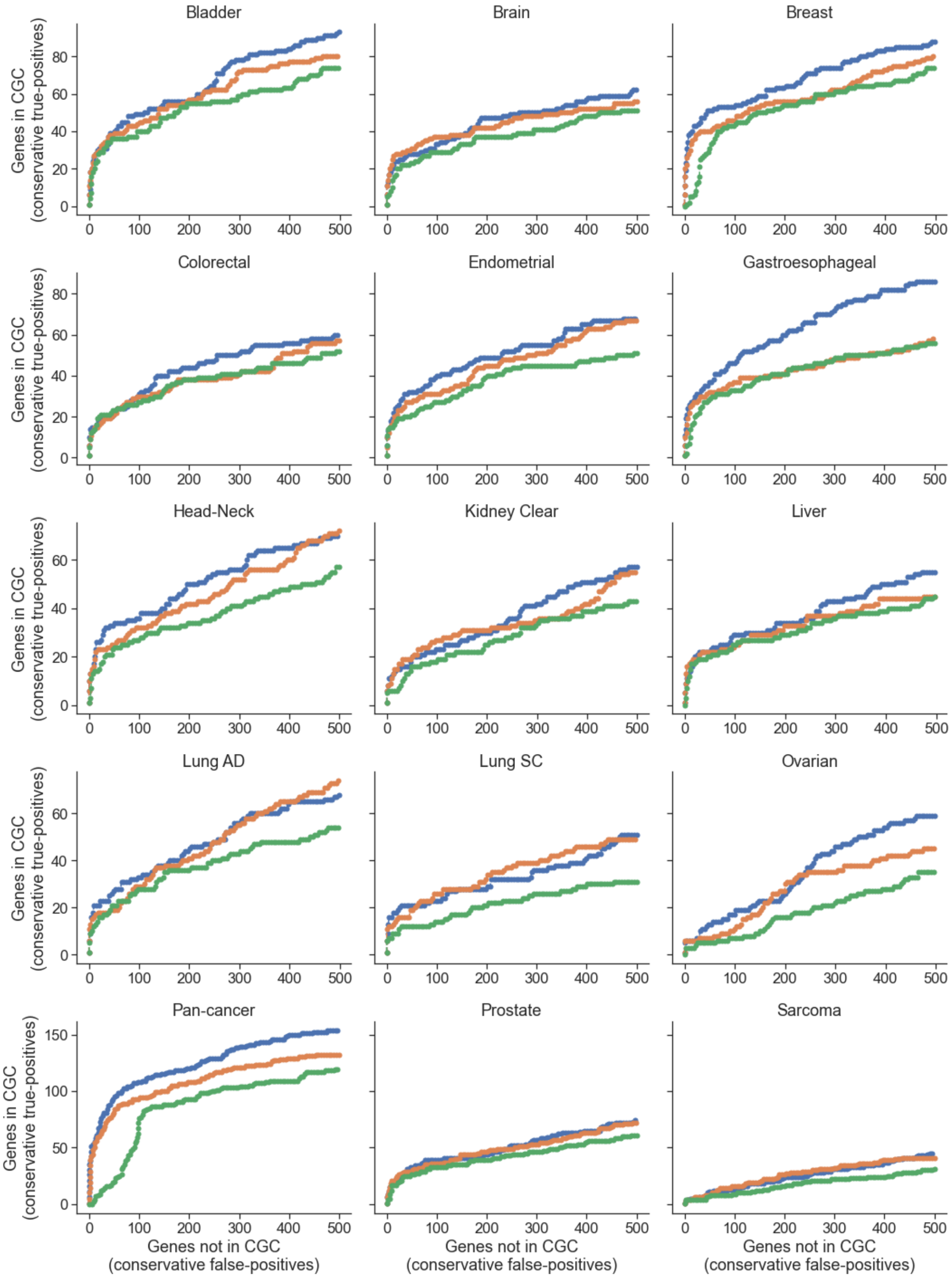

Supplementary figure 4: Approximate number of false-positive and true positive driver genes identified from 15 whole-exome sequenced cohorts from Dietlein et al.[4]. The numbers are approximate because the full set of driver genes is unknown; we therefore used genes in the CGC as a conservative approximation of true positives (since a non-CGC gene may still be a true driver). The MutSigCV model produced mis-calibrated p-values for the pan-cancer cohort, suggesting that its model assumptions may have been violated by the large cohort of heterogeneous cancer types.

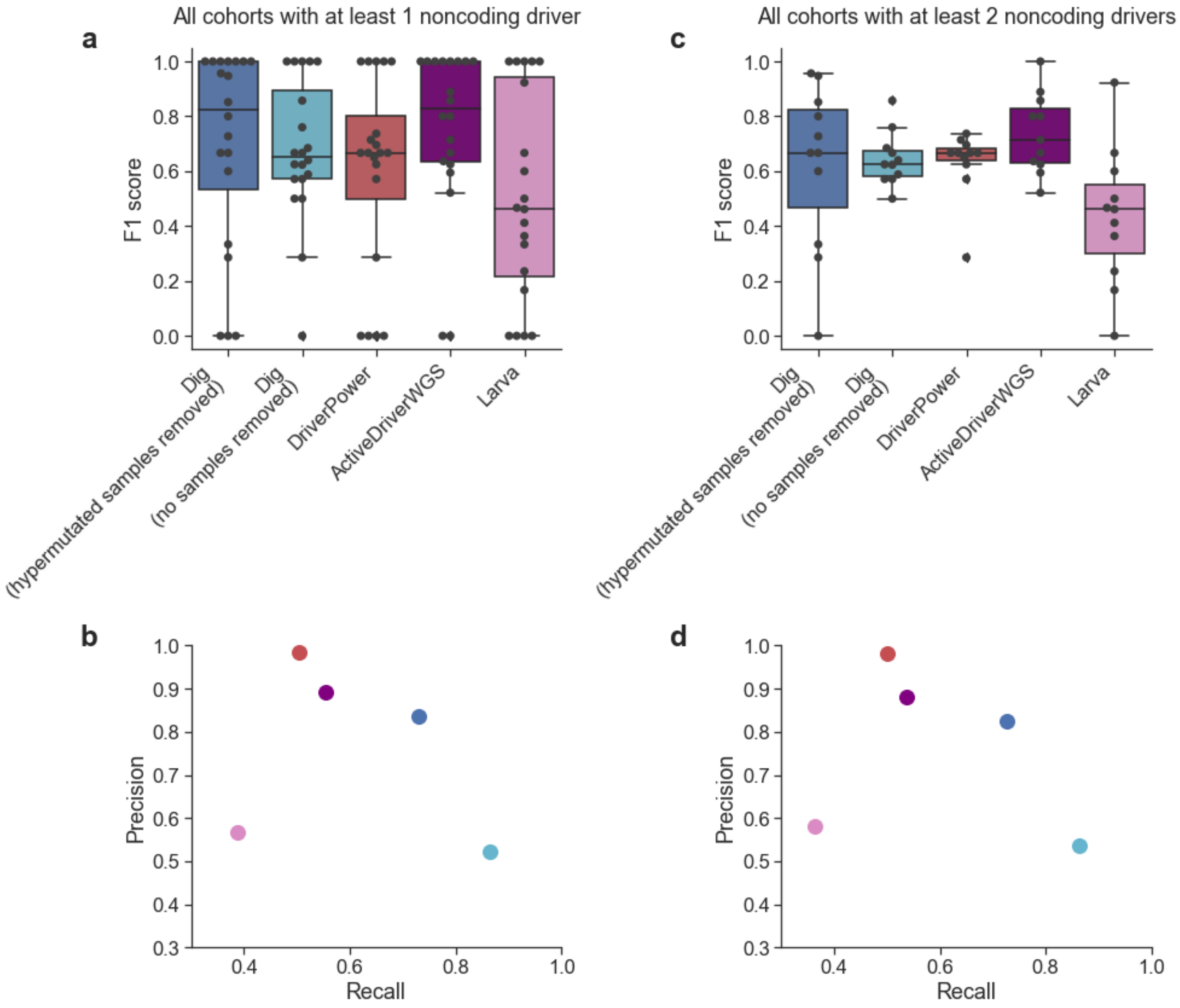

Supplementary figure 5: Precision-recall comparison of noncoding driver detection methods in the PCAWG dataset. a, F1-score across 95,231 noncoding elements as defined in Rheinbay et al.[10] in PCAWG cancer cohorts with at least one identified noncoding driver (n=20 cohorts). The performance of Dig was also evaluated when removing samples with >1000 SNVs across all elements and restricting the total number of SNVs per sample per element to 3. DriverPower and Larva do not have built-in filtering options. ActiveDriverWGS was run with default filtering which removes any sample with >30 SNVs per megabase. **b,** Recall and precision by method combined across the cohorts in **a**. **c,d,** as in **a** and **b** but restricting to n=11 cohorts with at least two identified noncoding drivers. Box-plot elements defined in Methods.

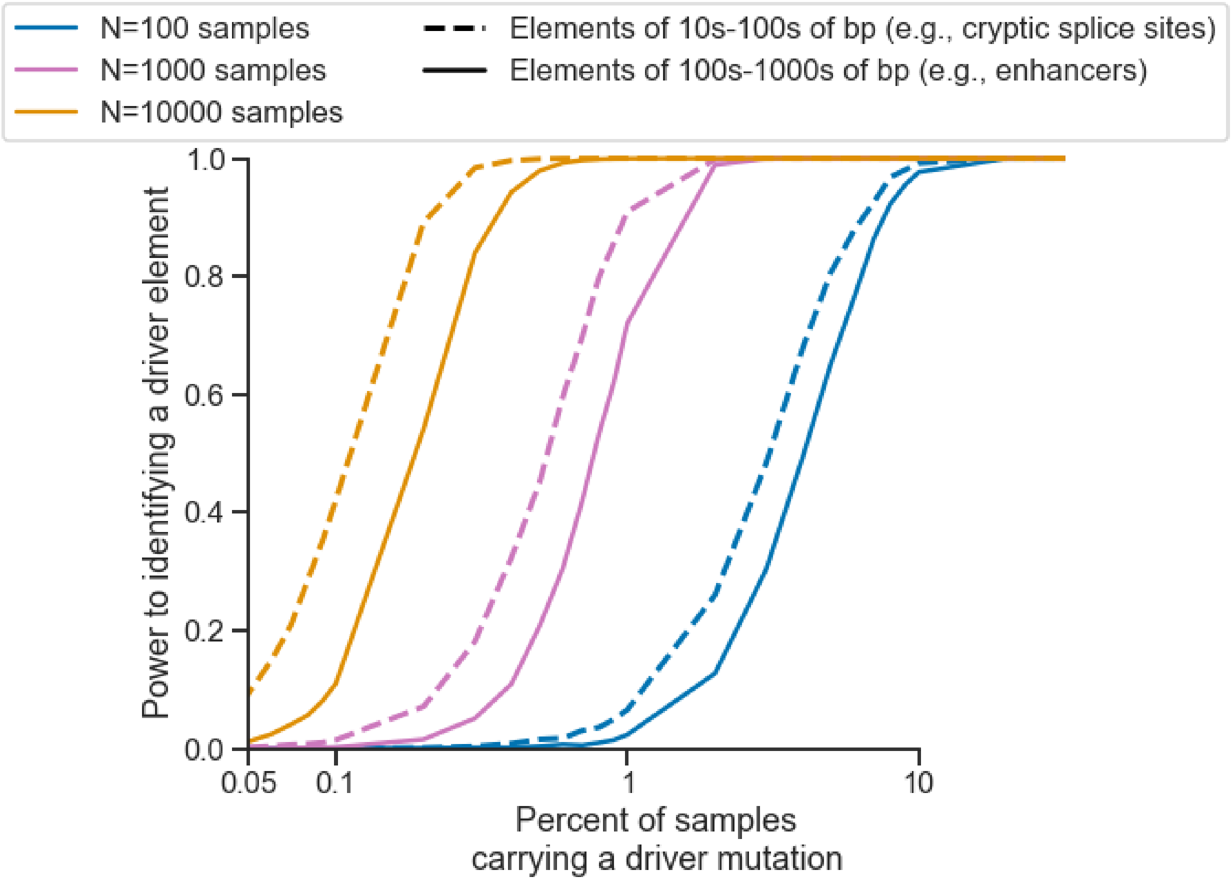

Supplementary Figure 6: Simulated power to detect driver elements in a pan-cancer cohort by sample size and by size of the elements being tested. The simulations were performed based on cryptic splice sites in 15,000 genes and 15,000 enhancers.

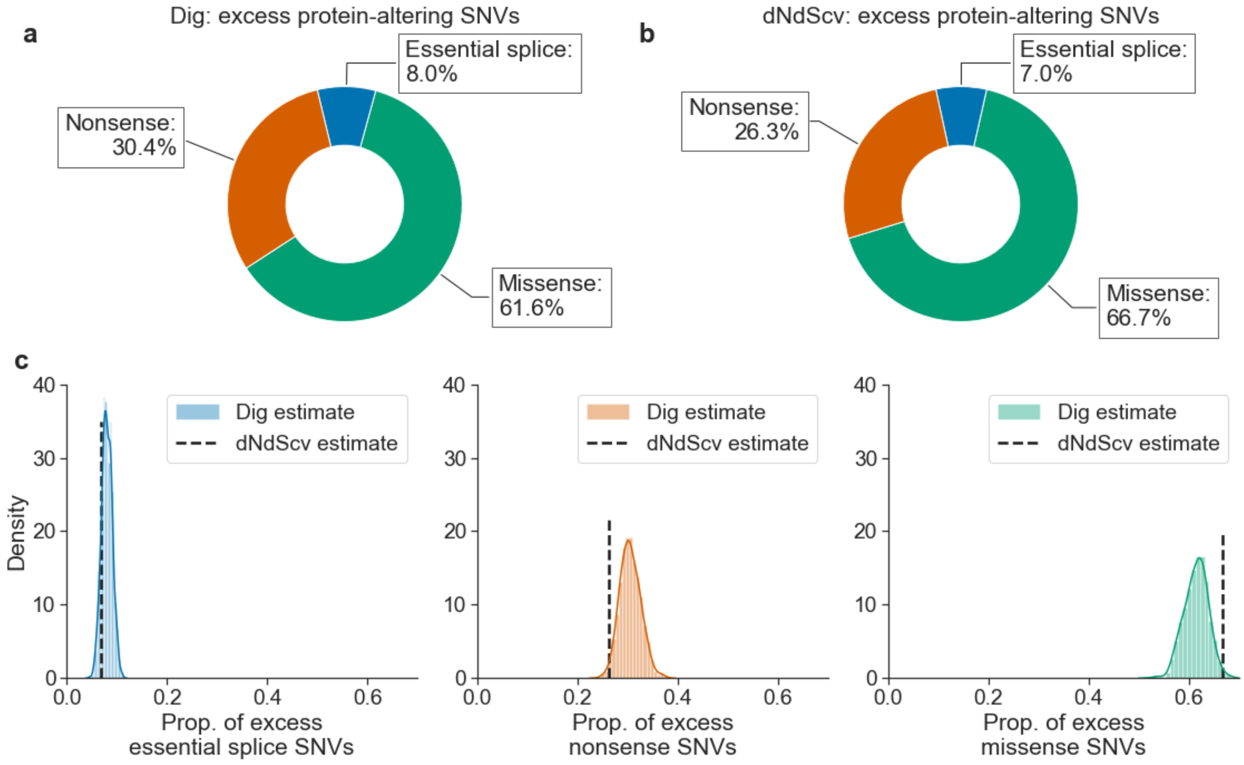

Supplementary figure 7: Proportion of excess protein-altering SNVs in TSGs as estimated by Dig, **a**, and dNdScv, **b**. **c,** Distribution of proportion of excess SNVs as estimated using a Monte Carlo simulation approach based on Dig (**Methods**) with the corresponding dNdScv estimate indicated with a black dashed line. Essential splice SNVs include SNVs at canonical splice sites (see **Fig. 3a**) and SNVs 5 bp 5' of an exon start, which dNdScv also considers in its analysis of splice mutations.

a

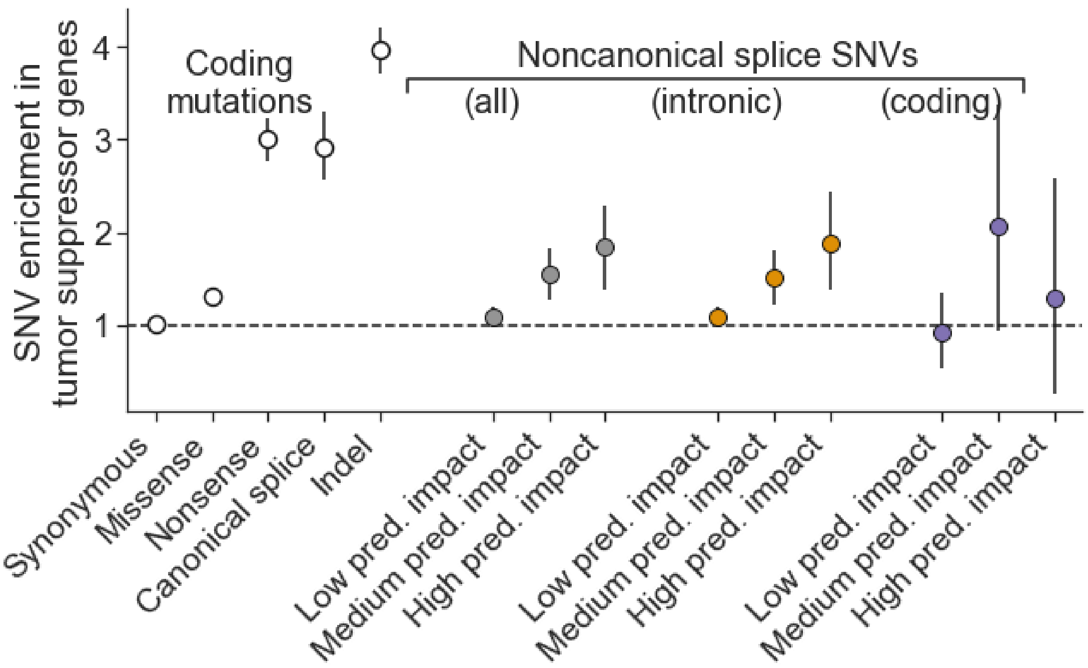

b

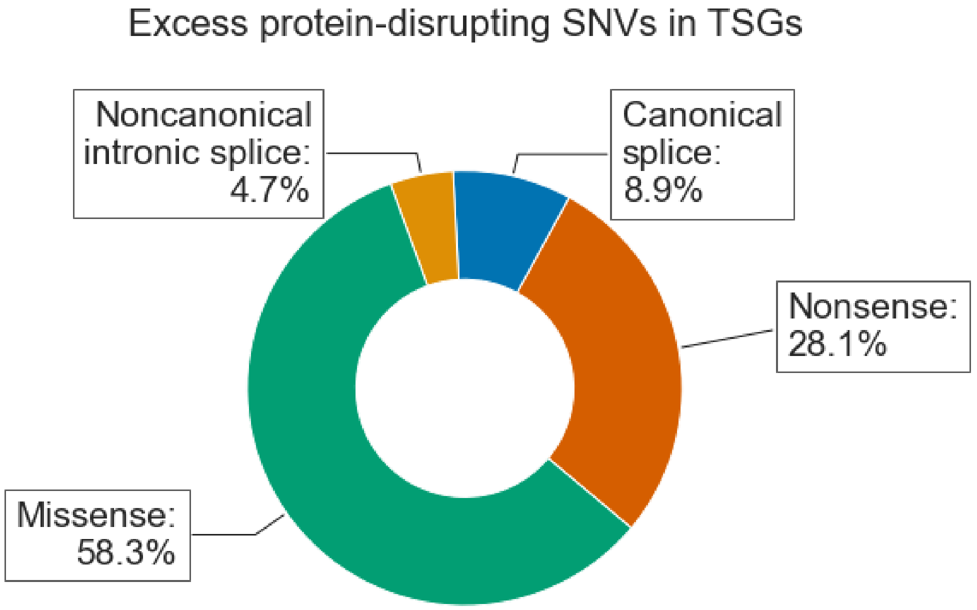

Supplementary figure 8: SNV enrichment (with 95% CI) and excess analysis excluding samples with >3000 coding mutations. a, as in **Fig. 3b** but excluding samples with >3000 coding mutations (default filtering criterion in dNdScv) (N=2,271 samples). **b,** As in **Fig. 3e** but excluding samples with >3000 coding mutations.

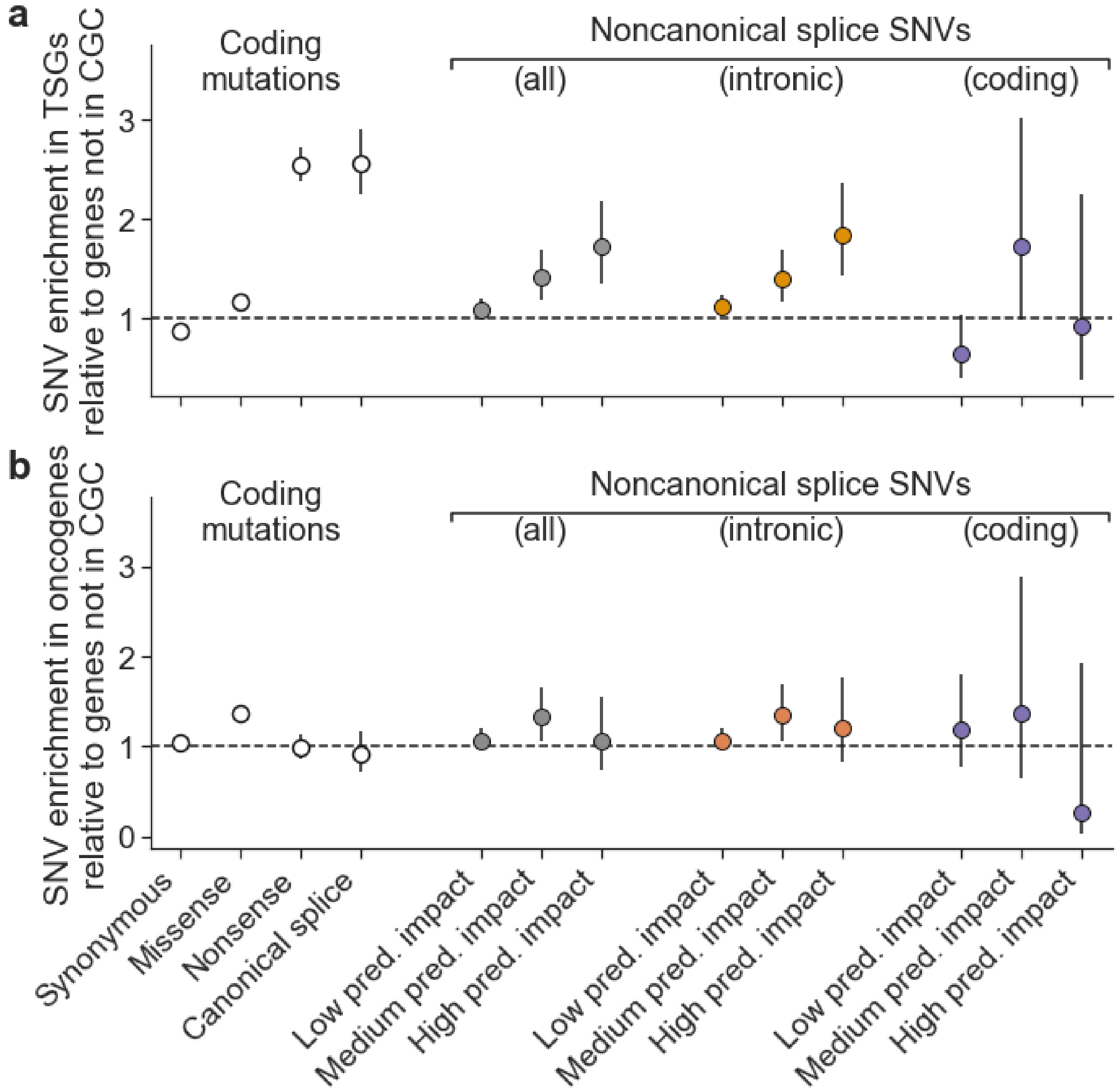

Supplementary figure 9: Estimated SNV enrichment with 95% CI in tumor suppressor genes (TSGs), **a**, and oncogenes, **b**, with enrichment calculated with respect to the number of observed mutations in genes not in the Cancer Gene Census (CGC). Enrichment is calculated as the rate of SNVs of a given type observed in TSGs (oncogenes) relative to the rate of SNVs of the same type observed in genes not in the CGC. (N=2,279 samples in both panels).

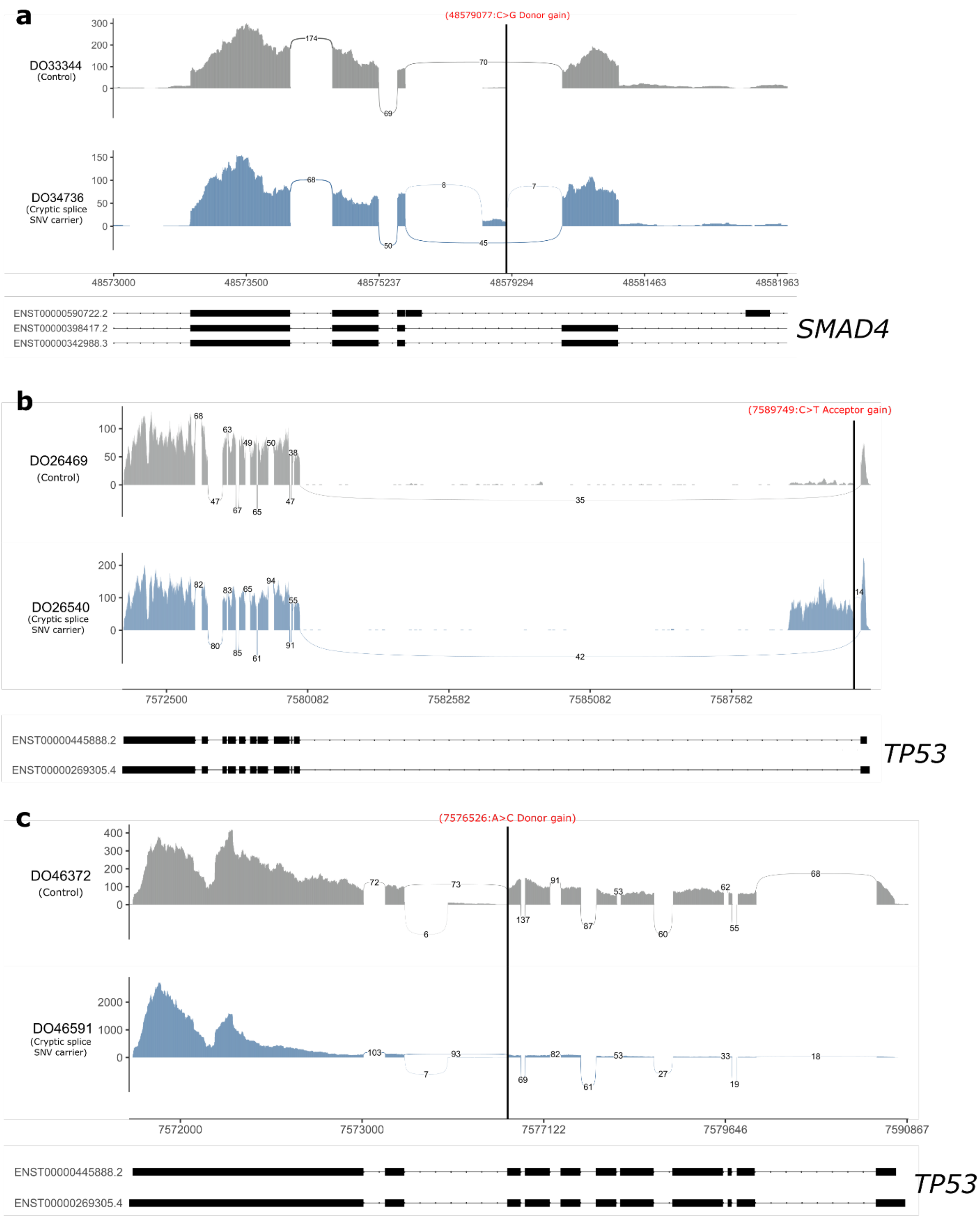

Supplementary Figure 10: Additional predicted cryptic splice SNV carriers in which LeafCutter identified strong evidence of alternative splicing. The location of the predicted cryptic splice SNV is marked with a thick black vertical line and labeled in red. **a,** *SMAD4* cryptic splice carrier. **b,c** *TP53* cryptic splice SNV carriers.

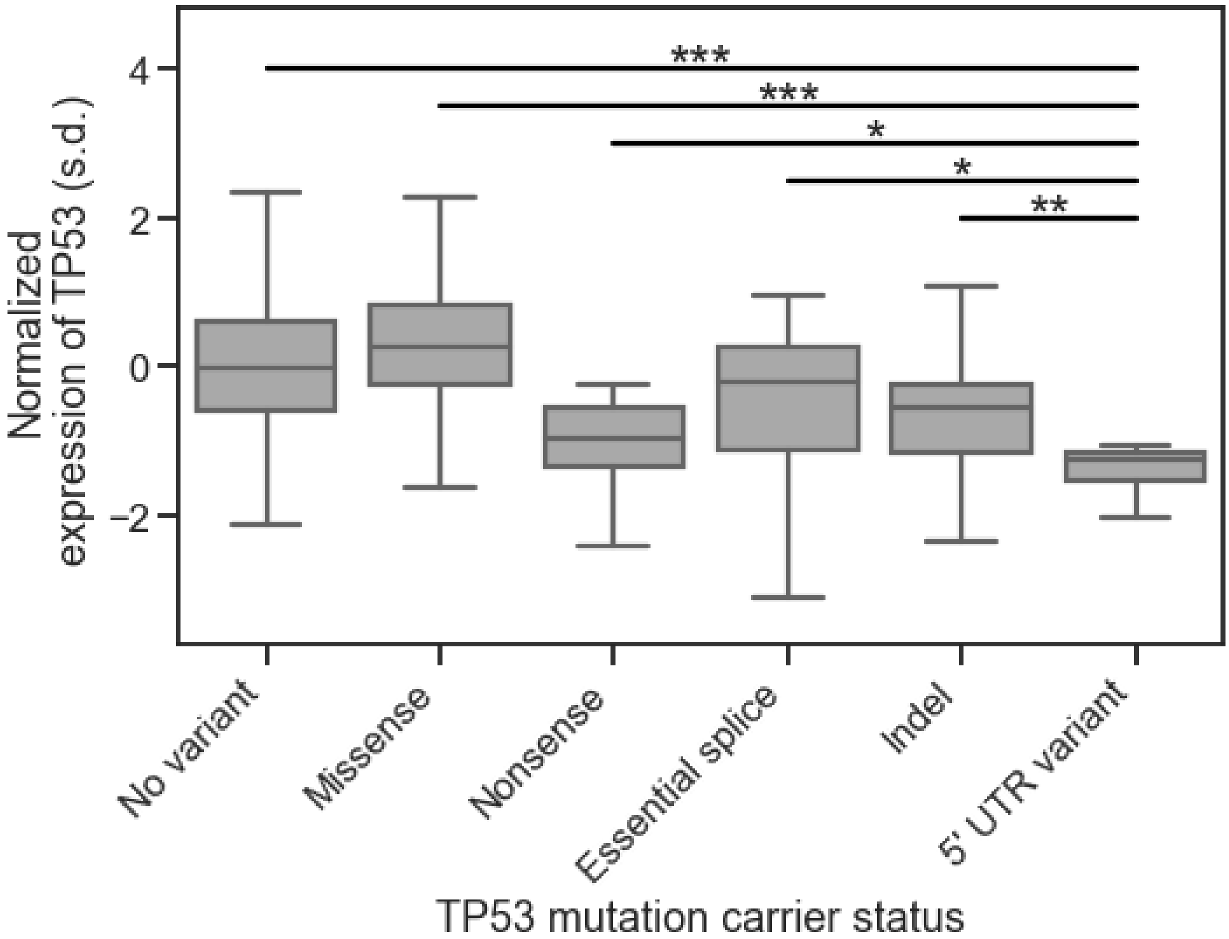

Supplementary Figure 11: **Normalized expression of TP53 stratified by the type of mutation individuals carry in TP53.** P-values comparing expression of 5' UTR variant carrier to other carrier categories: 5' UTR vs no variant: 1.2×10^{-4}; 5' UTR vs. missense: 3.3×10^{-5}; 5' UTR vs. nonsense: P=0.023; 5' UTR vs. essential splice: P=0.011; 5' UTR vs. coding indel: 8.5×10^{-3}. All p-values by one-sided Mann-Whitney U-test. (No variant: N=760 samples; Missense: N=285 samples; Nonsense: N=50 samples; Essential splice: N=35 samples; Indel: N=78 samples; 5' UTR variant: N=6 samples). Boxplot elements defined in Methods.

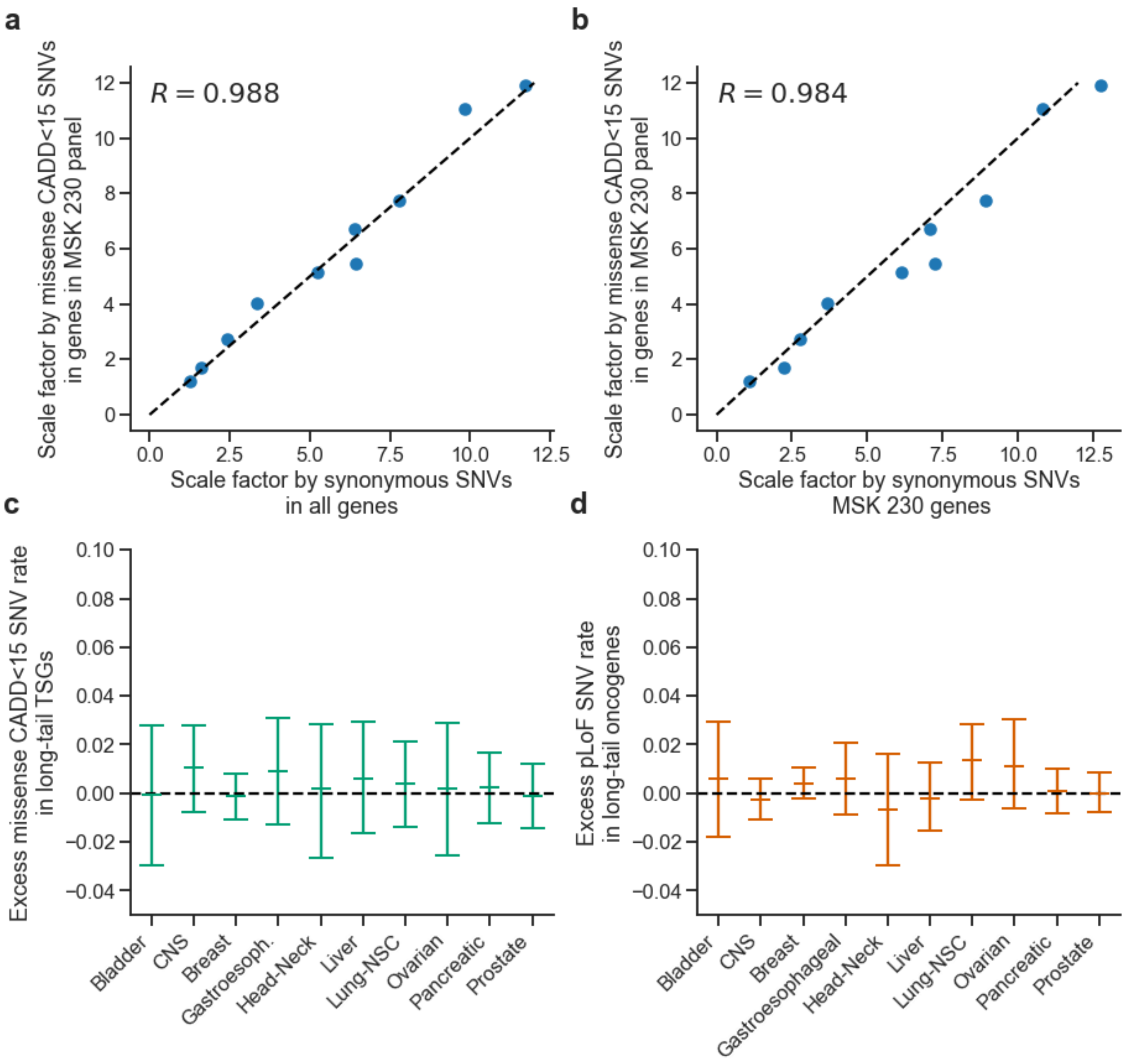

Supplementary figure 12: Evaluation of neutral mutation model for ten solid cancer megacohorts. Using whole-exome sequenced samples, we compared the accuracy of estimating the scaling factor based on missense SNVs with CADD phred<15 observed in genes in the MSK IMPACT 230 targeted sequencing panel (the approach used for analyzing the megacohorts, see Methods) to the scaling factor estimated using synonymous mutations observed in all autosomal genes (Dig's default method), **a**, and using synonymous mutations observed in genes in the MSK IMPACT 230 targeted sequencing panel, **b**. **c,** The estimated rate of excess missense SNVs with CADD phred<15 (with 95% CI) in tumor suppressor genes in the MSK IMPACT 230 targeted sequencing panel. The burden of missense SNVs with CADD phred<15 is not significant in any cancer type. **d,** The rate of excess pLoF SNVs in oncogenes (with 95% CI) in the MSK IMPACT 230 targeted sequencing panel. The burden of pLoF SNVs is not significant in any cancer type. N samples per cancer in **Supplementary Table 19** for **c** and **d**).

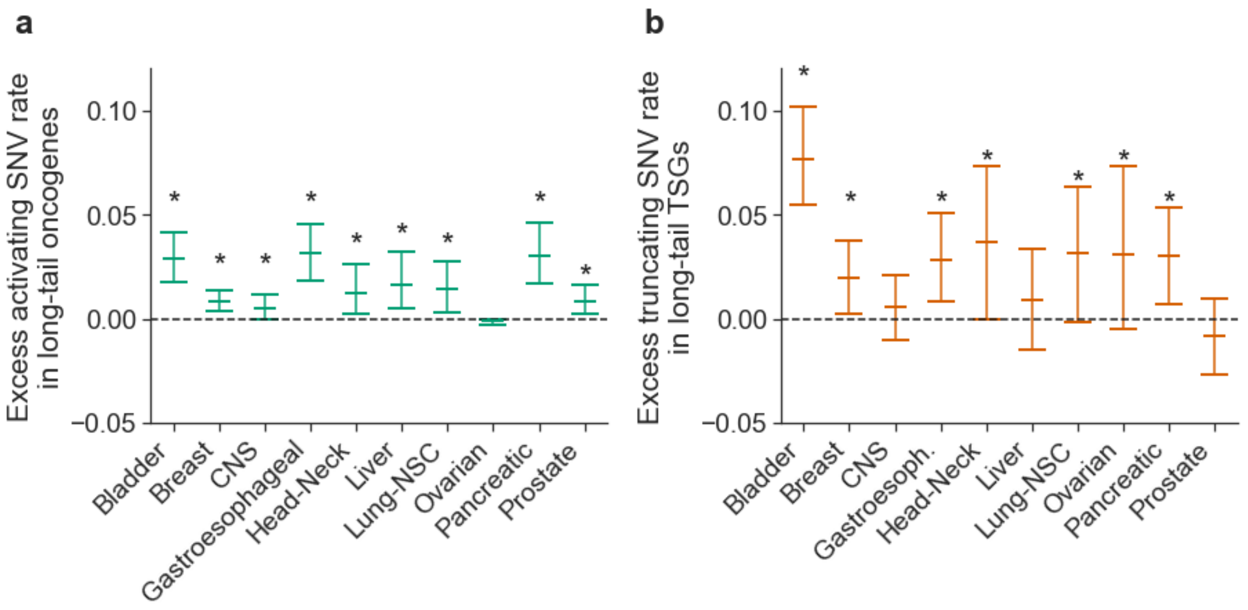

Supplementary figure 13: Estimated excess activating SNV rate in oncogenes with 95% CIs, **a**, and excess pLoF SNV rate in TSGs with 95% CIs, **b**, as in **Fig. 4a,b** but with analysis restricted to whole-exome sequenced samples only. Asterisks indicate the burden of SNVs is significant in the given cancer type. (N samples per cancer in **Supplementary Table 28**). Error bars are larger than in **Fig. 4a,b** because sample size is smaller (see **Supplementary Tables 22-23** for exact sample sizes).

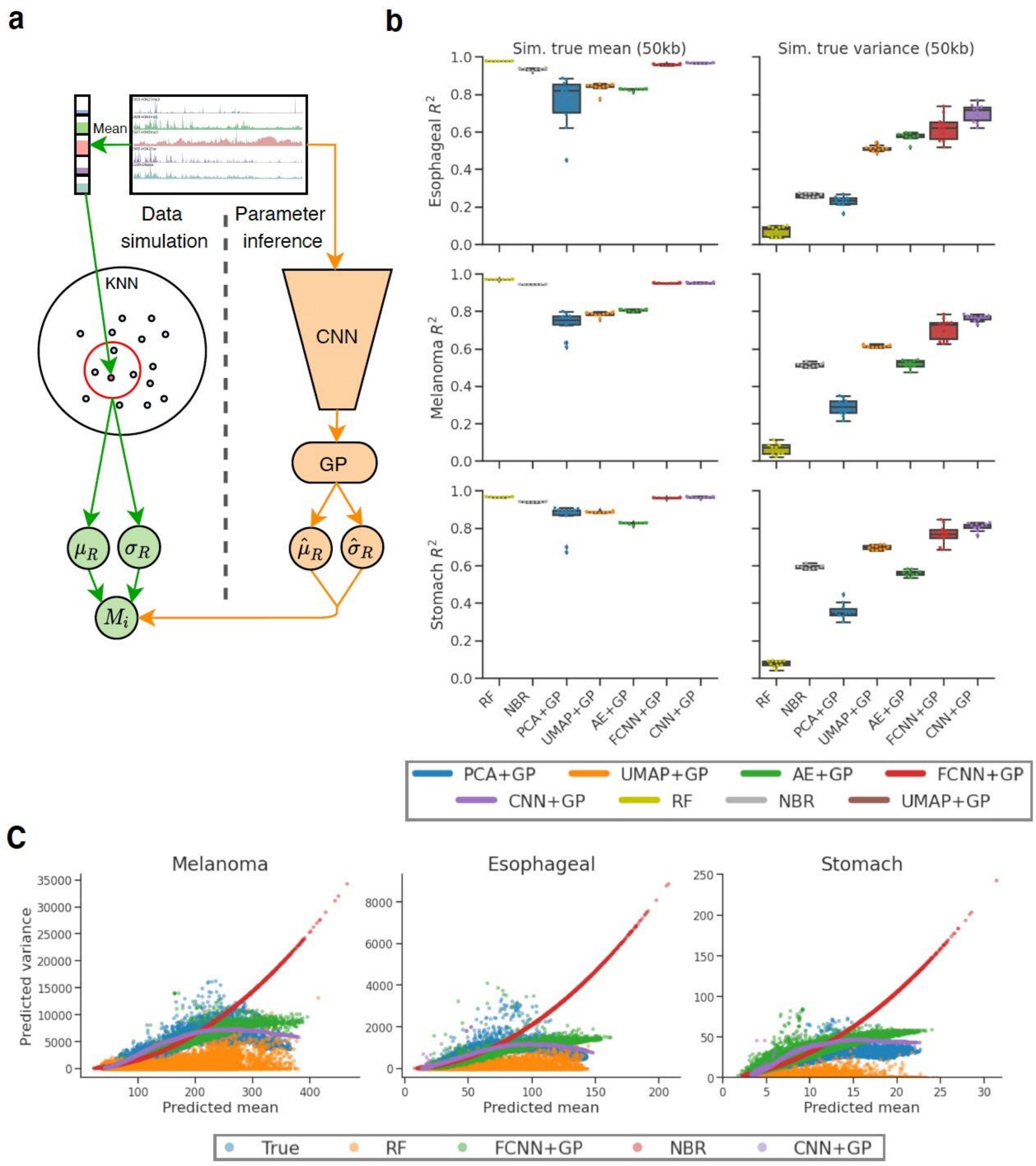

Supplementary figure 14: Comparison of mean and variance prediction accuracy by method in simulated datasets. a, Schematic of simulation framework. Briefly, for each 50kb region of the genome, a "true" mean and variance are constructed using a K-nearest-neighbors algorithm. The number of observed neutral mutations in that region is then simulated from a negative binomial distribution parameterized by this mean and variance. A method is then trained to predict the unknown mean and variance using the simulated number of mutations as a noisy objective. The accuracy of the model is evaluated by comparing the predicted mean and variance parameters to the known simulated values. **b,** Accuracy (Pearson's R^2) of the predicted

mean and variance compared to the true simulated mean and variance across methods (N=10 independent replicates per category). RF: random forest; NBR: negative binomial regression; PCA+GP: principle components dimensionality reduction following by a Gaussian process; UMAP+GP: UMAP dimensionality reduction followed by a Gaussian process; AE+GP: autoencoder dimensionality reduction followed by a Gaussian process; FCNN+GP: fully connected neural network followed by a Gaussian process; CNN+GP: Convolutional neural network followed by a Gaussian process (Dig's default model). Boxplot elements defined in Methods. **c,** Mean versus variance of the simulated data (blue) and predicted by a CNN+GP (purple), FCNN+GP (green), negative binomial regression (red), or a random forest (orange).

1. Paszke, A. *et al.* PyTorch: An Imperative Style, High-Performance Deep Learning Library. *arXiv:1912.01703 [cs, stat]* (2019).
2. Titsias, M. K. Variational Learning of Inducing Variables in Sparse Gaussian Processes. *AISTATS* 8 (2009).
3. Gardner, J. R., Pleiss, G., Bindel, D., Weinberger, K. Q. & Wilson, A. G. GPyTorch: Blackbox Matrix-Matrix Gaussian Process Inference with GPU Acceleration. *arXiv:1809.11165 [cs, stat]* (2019).
4. Dietlein, F. *et al.* Identification of cancer driver genes based on nucleotide context. *Nature Genetics* 1–11 (2020) doi:10.1038/s41588-019-0572-y.
5. Yaari, A. U. *et al.* Multi-resolution modeling of a discrete stochastic process identifies causes of cancer. in (2020).
6. Martincorena, I. *et al.* Universal Patterns of Selection in Cancer and Somatic Tissues. *Cell* **171**, 1029-1041.e21 (2017).
7. Alexandrov, L. B. *et al.* The repertoire of mutational signatures in human cancer. *Nature* **578**, 94–101 (2020).
8. Simonyan, K., Vedaldi, A. & Zisserman, A. Deep Inside Convolutional Networks: Visualising Image Classification Models and Saliency Maps. *arXiv:1312.6034 [cs]* (2014).
9. meuleman. *meuleman/epilogos*. (2021).
10. Rheinbay, E. *et al.* Analyses of non-coding somatic drivers in 2,658 cancer whole genomes. *Nature* **578**, 102–111 (2020).
11. Lawrence, M. S. *et al.* Mutational heterogeneity in cancer and the search for new cancer-associated genes. *Nature* **499**, 214–218 (2013).
12. Shuai, S., PCAWG Drivers and Functional Interpretation Working Group, Gallinger, S., Stein, L., & PCAWG Consortium. Combined burden and functional impact tests for cancer driver discovery using DriverPower. *Nat Commun* **11**, 734 (2020).

13. Lochovsky, L., Zhang, J., Fu, Y., Khurana, E. & Gerstein, M. LARVA: an integrative framework for large-scale analysis of recurrent variants in noncoding annotations. *Nucleic Acids Res* **43**, 8123–8134 (2015).
14. Kerpedjiev, P. *et al.* HiGlass: web-based visual exploration and analysis of genome interaction maps. *Genome Biology* **19**, 125 (2018).
15. Wu, J., Anczuków, O., Krainer, A. R., Zhang, M. Q. & Zhang, C. OLego: fast and sensitive mapping of spliced mRNA-Seq reads using small seeds. *Nucleic Acids Res* **41**, 5149–5163 (2013).
16. Cotto, K. C. *et al.* RegTools: Integrated analysis of genomic and transcriptomic data for the discovery of splicing variants in cancer. *bioRxiv* 436634 (2021) doi:10.1101/436634.
17. Li, Y. I. *et al.* Annotation-free quantification of RNA splicing using LeafCutter. *Nat Genet* **50**, 151–158 (2018).
18. Supek, F. & Lehner, B. Scales and mechanisms of somatic mutation rate variation across the human genome. *DNA Repair (Amst)* **81**, 102647 (2019).
19. Ching, T. *et al.* Opportunities and obstacles for deep learning in biology and medicine. *J R Soc Interface* **15**, 20170387 (2018).
20. Avsec, Ž. *et al.* Base-resolution models of transcription-factor binding reveal soft motif syntax. *Nat Genet* **53**, 354–366 (2021).
21. Hie, B., Zhong, E. D., Berger, B. & Bryson, B. Learning the language of viral evolution and escape. *Science* **371**, 284–288 (2021).
22. Tate, J. G. *et al.* COSMIC: the Catalogue Of Somatic Mutations In Cancer. *Nucleic Acids Research* **47**, D941–D947 (2019).
23. Zhu, H. *et al.* Candidate Cancer Driver Mutations in Distal Regulatory Elements and Long-Range Chromatin Interaction Networks. *Molecular Cell* **77**, 1307-1321.e10 (2020).
24. Karczewski, K. J. *et al.* The mutational constraint spectrum quantified from variation in 141,456 humans. *Nature* **581**, 434–443 (2020).

25. Kunkel, T. A. & Erie, D. A. Dna Mismatch Repair. *Annual Review of Biochemistry* **74**, 681–710 (2005).

26. Li, G.-M. Mechanisms and functions of DNA mismatch repair. *Cell Res* **18**, 85–98 (2008).

27. Kahles, A. *et al.* Comprehensive Analysis of Alternative Splicing Across Tumors from 8,705 Patients. *Cancer Cell* **34**, 211-224.e6 (2018).

28. Nasser, J. *et al.* Genome-wide enhancer maps link risk variants to disease genes. *Nature* **593**, 238–243 (2021).
