# Supplementary material for: Genome-wide mapping of somatic mutation rates uncovers drivers of cancer
Source: Nat Biotechnol. 2022 Jun 20;40(11):1634–43. doi: 10.1038/s41587-022-01353-8 (PMC9646522; doi:10.1038/s41587-022-01353-8)
Supplement: Supplementary file 2 — Reporting Summary [file 41587_2022_1353_MOESM2_ESM.pdf]

nature research

Corresponding author(s): Po-Ru Loh
Bonnie Berger

Last updated by author(s): Feb 17, 2022

Reporting Summary

Nature Research wishes to improve the reproducibility of the work that we publish. This form provides structure for consistency and transparency in reporting. For further information on Nature Research policies, see our Editorial Policies and the Editorial Policy Checklist.

Statistics

For all statistical analyses, confirm that the following items are present in the figure legend, table legend, main text, or Methods section.

n/a	Confirmed	
☐	☒	The exact sample size (*n*) for each experimental group/condition, given as a discrete number and unit of measurement
☐	☒	A statement on whether measurements were taken from distinct samples or whether the same sample was measured repeatedly
☐	☒	The statistical test(s) used AND whether they are one- or two-sided<br>*Only common tests should be described solely by name; describe more complex techniques in the Methods section.*
☐	☒	A description of all covariates tested
☐	☒	A description of any assumptions or corrections, such as tests of normality and adjustment for multiple comparisons
☐	☒	A full description of the statistical parameters including central tendency (e.g. means) or other basic estimates (e.g. regression coefficient) AND variation (e.g. standard deviation) or associated estimates of uncertainty (e.g. confidence intervals)
☐	☒	For null hypothesis testing, the test statistic (e.g. F, t, r) with confidence intervals, effect sizes, degrees of freedom and P value noted<br>*Give P values as exact values whenever suitable.*
☐	☒	For Bayesian analysis, information on the choice of priors and Markov chain Monte Carlo settings
☒	☐	For hierarchical and complex designs, identification of the appropriate level for tests and full reporting of outcomes
☐	☒	Estimates of effect sizes (e.g. Cohen's *d*, Pearson's *r*), indicating how they were calculated

Our web collection on statistics for biologists contains articles on many of the points above.

Software and code

Policy information about availability of computer code

Data collection	All data used were obtained from publicly available repositories and not software was used for data collection.
Data analysis	Dig v0.2 (https://github.com/maxwellsh/DIGDriver & https://anaconda.org/mutation_density/digdriver)<br>Python 3.7.6<br>MutSigCV v1.41<br>dNdScv v0.1<br>Larva v2.0<br>ActiveDriverWGS v1.1.1<br>DriverPower v1.0.2<br>bedtools v2.30<br>Olego v1.1.7<br>Regtools v0.5.2<br>LeafCutter v0.2.9<br>ggsashimi v.1.1.5<br>R

For manuscripts utilizing custom algorithms or software that are central to the research but not yet described in published literature, software must be made available to editors and reviewers. We strongly encourage code deposition in a community repository (e.g. GitHub). See the Nature Research guidelines for submitting code & software for further information.

Data

Policy information about availability of data

All manuscripts must include a data availability statement. This statement should provide the following information, where applicable:

- Accession codes, unique identifiers, or web links for publicly available datasets
- A list of figures that have associated raw data
- A description of any restrictions on data availability

Data generated as part of this study are available as supplementary tables or from http://dig-cancer.csail.mit.edu/. Browsable mutation maps for 37 cancer types are provided at https://resgen.io/maxsh/Cancer_Mutation_Maps/views.
PCAWG data are available from https://dcc.icgc.org/releases/PCAWG/.
Hartwig Medical Foundation data are available from https://database.hartwigmedicalfoundation.nl/.
Whole exome sequencing data compiled by Dietlein et al. are available from http://www.cancer-genes.org/.
Targeted sequencing data are available from https://www.cbioportal.org/.
The list of genes in the Cancer Gene Census are available at https://cancer.sanger.ac.uk/cosmic/download.
The gnomad dataset is available from https://gnomad.broadinstitute.org/downloads

Field-specific reporting

Please select the one below that is the best fit for your research. If you are not sure, read the appropriate sections before making your selection.

☒ Life sciences ☐ Behavioural & social sciences ☐ Ecological, evolutionary & environmental sciences

For a reference copy of the document with all sections, see nature.com/documents/nr-reporting-summary-flat.pdf

Life sciences study design

All studies must disclose on these points even when the disclosure is negative.

Sample size	Sample size was determined by the total number of samples available for a given cancer type from the publicly available resources used in this study. No method was used to predetermine sample size.
Data exclusions	Where explicitly stated, we removed samples with >3000 somatic coding mutations. This is a standard filtration procedure applied in numerous prior works to exclude samples with possible hypermutation processes.
Replication	Where explicitly stated in the manuscript, we verified that mutational patterns observed in the Pan-Cancer Analysis of Whole Genomes dataset were also observed in the Hartwig Medical Foundation dataset by downloading mutations from the Hartwig Medical Foundation dataset for the element of interest. For the ELF3 5' UTR, we calculated the enrichment of observed mutations to expected mutations and compared this to the enrichment calculated for the same regions in the PCAWG dataset. All attempts at replication were successful.
Randomization	Samples were allocated into groups based on tumor type, which was predetermined and provided in the public repositories.
Blinding	Investigators were not blind to group allocation because groups were predetermined by tumor type.

Reporting for specific materials, systems and methods

We require information from authors about some types of materials, experimental systems and methods used in many studies. Here, indicate whether each material, system or method listed is relevant to your study. If you are not sure if a list item applies to your research, read the appropriate section before selecting a response.

Materials & experimental systems

n/a	Involved in the study	
☒	☐	Antibodies
☒	☐	Eukaryotic cell lines
☒	☐	Palaeontology and archaeology
☒	☐	Animals and other organisms
☒	☐	Human research participants
☒	☐	Clinical data
☒	☐	Dual use research of concern

Methods

n/a	Involved in the study	
☒	☐	ChIP-seq
☒	☐	Flow cytometry
☒	☐	MRI-based neuroimaging
